# Concise Six-Step Asymmetric Approach to Ramelteon from an Acetophenone Derivative Using Ir, Rh, Cu, and Ni Catalysis

**DOI:** 10.1021/acs.joc.1c01614

**Published:** 2021-09-30

**Authors:** Jérôme Cluzeau, Ulrike Nettekoven, Miroslav Planinc Kovačevič, Zdenko Časar

**Affiliations:** † Lek Pharmaceuticals d.d., Sandoz Development Center Slovenia, Kolodvorska 27, 1234 Mengeš, Slovenia; ‡ Solvias AG, Römerpark 2, 4303 Kaiseraugst, Switzerland; § University of Ljubljana, Faculty of Pharmacy, Aškerčeva c. 7, SI-1000 Ljubljana, Slovenia

## Abstract

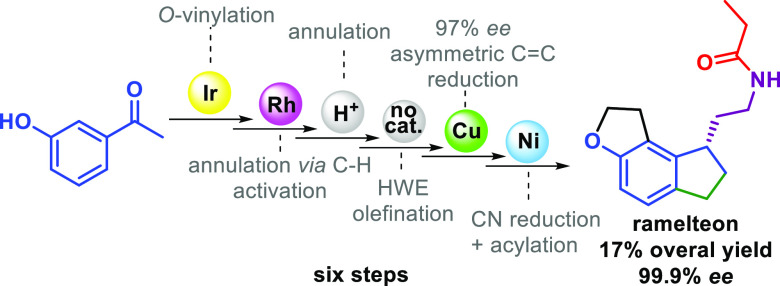

A concise six-step asymmetric synthesis of nearly enantiomerically pure ramelteon was developed from a monocyclic precursor with a 17% overall yield and a 97% ee in the asymmetric step. The synthetically challenging tricyclic 1,6,7,8-tetrahydro-2*H*-indeno[5,4-*b*]furan core of ramelteon was assembled by using Ir-catalyzed *O*-vinylation and Rh-catalyzed vinyl ether annulation through directed C–H bond activation, while the chirality was introduced with enantioselective reduction of an α,β-unsaturated nitrile moiety under hydrosilylation conditions using a Cu^II^/Walphos type catalyst. The presented methodology represents the shortest synthetic approach to ramelteon.

Ramelteon ((*S*)-*N*-[2-(1,6,7,8-tetrahydro-2*H*-indeno-[5,4-*b*]furan-8-yl)ethyl]propionamide; also known as TAK-375) was the first selective melatonin receptor (MT1 and MT2) agonist that was approved for the treatment of insomnia characterized by difficulty with sleep onset.^1^ The ramelteon market value in 2019 accounted for $161 million (USD), which makes it a valuable drug in the category of medications for the treatment of sleeping disorders. Moreover, in a recent clinical study, ramelteon was found to be effective for the prevention of delirium in elderly patients undergoing gastrectomy.^2^ Therefore, ramelteon represents an interesting synthetic target. However, ramelteon’s molecular structure consisting of a tricyclic 1,6,7,8-tetrahydro-2*H*-indeno[5,4-*b*]furan core containing a chiral substituent suggests that its concise and efficient asymmetric synthesis might be challenging. Although several synthetic approaches to ramelteon^3−7^ or its key intermediates^8−11^ have been devised, they have been either very long, starting from advanced bicyclic or tricyclic intermediates, or produced racemic ramelteon (Scheme 1, see Supporting Information document for expanded discussion). In our work, we present a concise six-step asymmetric synthesis of ramelteon from a monocyclic 3-hydroxyacetophenone starting material by using iridium, rhodium, copper, and nickel catalysis to construct the tricyclic core and assemble the chiral side chain.^12^ This work demonstrates the versatility and power of transition-metal catalysis in the total synthesis of demanding organic scaffolds in a concise and asymmetric fashion.

**Scheme 1:**
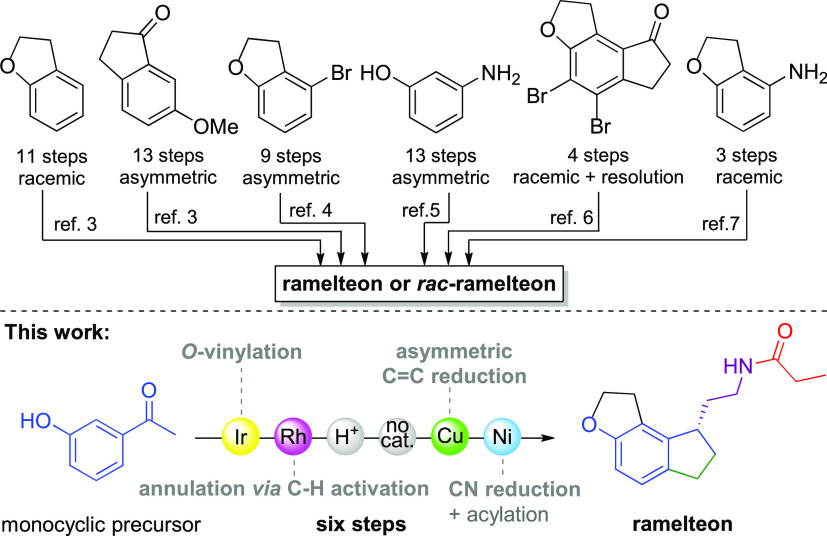
Previously Known Synthetic Strategies to Ramelteon and Our Approach

In order to develop a low process intensity synthesis of ramelteon, our goal was to devise a very concise asymmetric route starting from a simple monocyclic precursor. Thus, we have selected 3-hydroxyacetophenone **1**, which is a cheap and readily available commodity chemical, as a starting material for the synthesis of ramelteon. Based on the selected starting material **1**, it was obvious that several carbon–carbon and carbon–heteroatom bond-forming reactions will need to be performed in order to construct first the ramelteon tricyclic core and attach the chiral side chain. In the past, the homogeneous transition-metal-catalyzed reaction proved a powerful synthetic tool for such purpose.^13^ Therefore, in the first step of our synthetic approach, acetophenone **1** was subjected to Ishii’s *O*-vinylation with vinyl acetate **2** using the [Ir(cod)Cl]_2_ catalyst in the presence of Na_2_CO_3_ (Scheme 2).^14^ The reaction proceeded smoothly on a gram scale and provided full conversion with 1 mol % [Ir(cod)Cl]_2_ and 0.6 equiv of Na_2_CO_3_ after 2 h at 100 °C in toluene, giving the corresponding vinyl ether **3** in 85% yield. Subsequent experiments revealed that 1 mol % of [Ir(cod)Cl]_2_ is indeed required to perform the reaction, as 0.5 mol % of [Ir(cod)Cl]_2_ afforded only ca. 30% conversion of **1** to **3**. In the second step, the vinylic ether **3** was subjected to an intramolecular *ortho*-C–H bond activation/olefin insertion reaction^15^ to provide a 2,3-dihydrobenzofuran scaffold. Therefore, when the vinylic ether **3** was reacted with benzylamine in the presence of 4 Å molecular sieves in toluene at reflux, the corresponding aromatic imine was formed *in situ* in 18 h. The formed imine directing group activated the alkenyl group tethered at the *meta* position, which then underwent *ortho*-alkylation cyclization in the presence of Wilkinson’s catalyst (3 mol %) in toluene in 18 h at 130 °C to provide 1-(2,3-dihydrobenzofuran-4-yl)ethan-1-one **4** in 90% yield after the acidic workup (Scheme 2). Subsequently, 2,3-dihydrobenzofuran **4** was subjected to another annulation in order to form the cyclopentanone ring and thus ramelteon’s tricyclic 1,6,7,8-tetrahydro-2*H*-indeno[5,4-*b*]furan core **5**. For this purpose, we considered using an α-methylenation^16,17^/Nazarov cyclization^18^ sequence (Scheme 2). Indeed, it is known that acrylophenones are prone to undergo cyclization to indanones under acidic conditions.^19−21^ Therefore, acetophenone derivative **4** was subjected to α-methylenation using paraformaldehyde in the presence of *i*-Pr_2_NH·TFA salt in dry dioxane at reflux for 48 h. This afforded the acrylophenone intermediate that subsequently underwent cyclization in the presence of sulfuric acid solution (98% aq) at 40 °C to provide the desired 1,2,6,7-tetrahydro-8*H*-indeno[5,4-*b*]furan-8-one **5** in 27% isolated yield along with 35% of unreacted 2,3-dihydrobenzofuran **4** that can be reused in the next batch, which gives 41% yield of **5** based on the consumed **4**. Some reaction optimization experiments showed that **5** can be obtained in 39% isolated yield without unreacted **4** being present, if the α-methylenation reaction was performed in a closed system with the rigorous exclusion of water and the α,β-unsaturated ketone intermediate was added very slowly overnight to the sulfuric acid solution to prevent the formation of the biphasic system. Although the whole three-step synthetic sequence from **1** to **5** has a 31% yield, it is the shortest known route to **5** to date from a simple monocyclic precursor. Therefore, it still might be considered preferable compared to previously known routes to **5**, where 8 steps were used with an overall 30% yield in the primary synthetic route starting from bicyclic 2,3-dihydrobenzofuran,^3a,3b^ 5 steps were needed (41% yield) starting from an advanced bicyclic 6-methoxyindan-1-one precursor,^8^ or 7 steps were required (50% yield) using bromophenol as a starting material.^10^

**Scheme 2:**
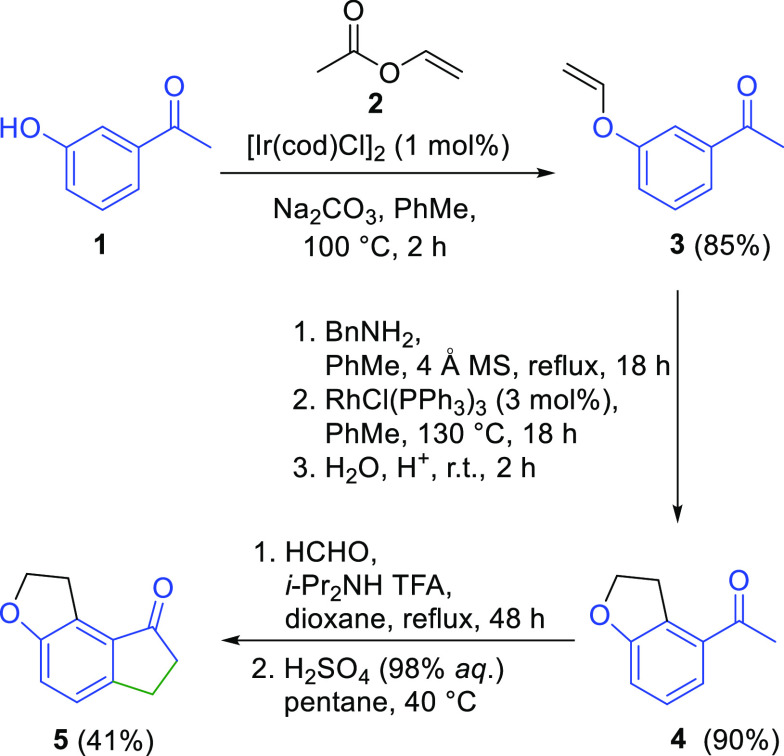
Synthesis of the Tricyclic 1,6,7,8-Tetrahydro-2*H*-indeno[5,4-*b*]furan Core of Ramelteon

After assembly of ramelteon’s tricyclic core **5**, we have focused our attention on the construction of a ramelteon amide functional group containing side chain, which is attached to the ramelteon’s tricyclic core via the stereogenic center. Therefore, in the next step, the ketone **5** was reacted under Horner–Wadsworth–Emmons olefination reaction conditions with diethyl (cyanomethyl)phosphonate **6** to provide α,β-unsaturated nitrile derivative **7** in 77% yield (Scheme 3).^3a−3c^ Although known asymmetric synthetic routes to ramelteon that use nitrile derivative **7** proceed subsequently via its conversion to the corresponding allylic acylamine or allylamine derivative^1a,3^ followed by an asymmetric reduction of the carbon–carbon double bond with Ru-BINAP or Rh-JosiPhos catalysts, we have decided to perform the asymmetric reduction before the manipulation of the nitrile group. For this purpose, we have chosen to explore Yun’s conjugate reduction using the Cu(II)/ligand catalytic system under hydrosilylation conditions, which proved very efficient in highly enantioselective reductions of acyclic aryl-substituted α,β-unsaturated nitriles with Cu(OAc)_2_/JosiPhos catalysts.^22^ Our initial scouting experiment of conjugate reduction of α,β-unsaturated nitrile **7** (Scheme 3) was performed on a 2 mmol scale with JosiPhos-type (*S*)-1-[(*R*)-2-(diphenylphosphino)ferrocenyl]ethyldicyclohexylphosphine ligand (enantiomer of **L4** in Scheme 3) and Cu(OAc)_2_ as a metal precursor (S/C = 25, 1.1 equiv of ligand/metal) in PhMe, *t*-BuOH, and CH_2_Cl_2_ (4.0:0.8:0.2, v/v) at room temperature in the presence of polymethylhydrosiloxane (PMHS) for 5 h. Under these reaction conditions, promising 95% conversion of **7** to **8** and in good enantioselectivity of 83% ee (*S*) was obtained. In order to determine the optimal catalytic system at a minimal catalyst load, we performed additional catalysts screening. To maximize the experimental efficiency and rapidly assess the optimal catalytic system for the conjugate reduction of α,β-unsaturated nitrile **7**, we employed a high-throughput experimentation^23^ to evaluate a large collection of ligands. Thus, the high-throughput screening (HTS) of the desired transformation was conducted on an 83 μmol scale of **7** using in total 1 metal precursor [Cu(OAc)_2_], 48 different ligands from diverse ligand families (BINAP,^24^ BDPP,^25^ MeOBIPHEP,^26^ Phosferrox,^27^ TaniaPhos,^28^ MonoPhos,^29^ DifluorPhos,^30^ PhanePhos,^31^ JoSPOphos,^32^ MandyPhos,^33^ JosiPhos,^34^ WalPhos,^35^ and ChenPhos,^36^), S/C ratios of 25 and 100, in the presence of polymethylhydrosiloxane (PMHS), toluene, and dichloroethane as solvents, in overall 96 experiments on the reaction plate. Reactions conditions were chosen: room temperature, argon atmosphere, and 20 h (Scheme 3, see the Supporting Information for details). Results of the conducted HTS revealed that the highest levels of enantioselectivity for conversion of **7** to **8** were observed with the use of selected MandyPhos, JosiPhos, and WalPhos type ligands **L1**–**L7** (Scheme 3, Table 1). The best performance in terms of enantioselectivity was obtained with catalysts based on WalPhos-type ligands **L1−L3** (Scheme 3, Table 1, entries 1–4), where ca. 87–96% ee values were attained. It is worth noting that among **L1**–**L3** ligands **L2** and **L3** gave practically full conversion (99.1–100%) and nearly complete selectivity (99–100%) with only minimal amounts of side products formed (Table 1, entries 2–4), whereas **L1** afforded 87% conversion along with ca. 8% of side products. Interestingly, catalysts based on ligand **L3** performed in nearly the same way at S/C of 25 and 100 in the context of conversion and selectivity but gave marginally higher enantioselectivity at a higher catalyst load (Table 1, entries 3 and 4). A similar performance was observed for catalysts based on ligand **L4** in the context of conversion and selectivity, but it gave marginally higher enantioselectivity at a lower catalyst load (Table 1, entries 5 and 6). The best overall performance with the catalysts based on the WalPhos family of ligands was observed with (*S*)-1-{(*S*_P_)-2-[2-[bis(4-methoxy-3,5-dimethylphenyl)phosphino]phenyl]ferrocenyl}ethylbis[3,5-bis(trifluoromethyl)phenyl]phosphine (**L2**), where 100% conversion, 99% selectivity, and 95.7% ee were obtained at S/C = 100. Next, catalysts based on JosiPhos-type ligands **L4**–**L6** gave 75–82% ee and nearly complete conversions (99.5–100%) and selectivities (98.7–100%) at both S/C ratios of 25 and 100 (Table 1, entries 5–9). The best performance in the JosiPhos family of ligands was obtained with **L4** at S/C = 100, where full conversion, complete selectivity, and 82% ee were obtained. Interesting results were also achieved with a catalyst based on MandyPhos ligand **L7**, which provided enantioselectivities in a range of 86% to 88% ee at S/C of 25 and 100, respectively (Table 1, entries 10 and 11). At both levels of catalyst loading, full selectivity was observed, while conversion dropped from 100% in the case of S/C = 25 to 89.5% for S/C = 100.

**Scheme 3:**
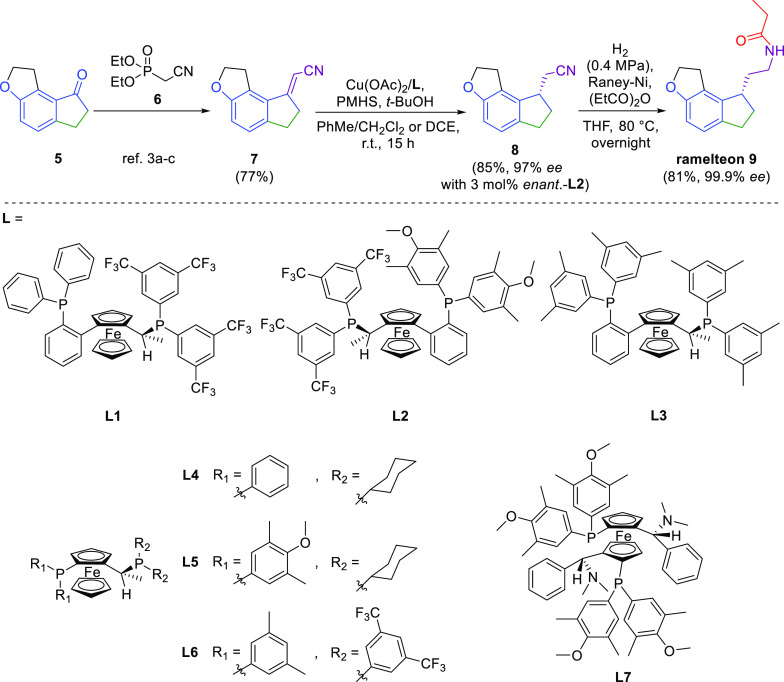
Formation of the Chiral Side Chain of Ramelteon

**Table 1:** Key Results of the High-Throughput Screening of Enantioselective Reduction of an α,β-Unsaturated Nitrile Moiety under Hydrosilylation Conditions Using Cu(OAc)_2_/Josiphos-Type/Related Catalysts

entry	ligand	S/C	conversion [%]^a^	ee [%]^a^	selectivity [%]^a^
1	**L1**	25	86.6	96.0	91.6
2	**L2**	100	100.0	–95.7	99.0
3	**L3**	25	100.0	90.6	100.0
4	**L3**	100	99.1	86.6	99.7
5	**L4**	25	100.0	–79.9	100.0
6	**L4**	100	100.0	–82.1	100.0
7	**L5**	25	100.0	–77.8	100.0
8	**L5**	100	99.5	–76.0	99.8
9	**L6**	25	100.0	–75.3	98.7
10	**L7**	25	100.0	86.0	100.0
11	**L7**	100	89.5	87.8	100.0

a Determined with supercritical fluid chromatography (see Experimental Section). Absolute configuration: “–“ denotes (*R*)-product in excess. Selectivity is defined as the difference between formed product **8** and other side products.

After the completion of high-throughput screening, an additional verification experiment was performed at ca. 1 mmol scale of **7** using the enantiomer of **L2**: (*R*)-1-{(*R*_P_)-2-[2-[bis(4-methoxy-3,5-dimethylphenyl)phosphino]phenyl]ferrocenyl}ethylbis[3,5-bis(trifluoromethyl)phenyl]phosphine with S/C = 33.3 (1.0 equiv of the ligand/metal ratio) in the presence of PMHS (4 equiv). The reaction was performed over 15 h in a solvent consisting of PhMe, *t*-BuOH, and CH_2_Cl_2_ (1.0:0.2:0.1) at room temperature under a nitrogen atmosphere. The reaction gave 83% conversion of **7** to **8** and allowed us to isolate the desired nitrile **8** in 72% yield and 97% ee along with 16% of unreacted **7**, which could be reused in the next nitrile **7** reduction batch. Thus, the yield of **8** based on the consumed **7** was 85%. Noteworthy, a similar reaction performed at 1 mmol scale under an argon atmosphere for 20 h in a solvent consisting of PhMe, *t*-BuOH, and CH_2_Cl_2_ (1.0:0.2:0.05) at S/C = 100 (1.1 equiv of the ligand/metal ratio) provided 98% conversion along with 93% ee. Therefore, we believe that further fine-tuning of the reaction conditions could provide high conversion and optimal enantioselectivity.

In the final step, nitrile **8** was reduced by hydrogen (0.4 MPa) in the presence of Raney nickel in THF at 80 °C overnight to the corresponding amine and reacted simultaneously in the reaction mixture with propionic anhydride to form the corresponding amide functionality. This provided ramelteon, which was isolated after crystallization from the AcOEt/hexane mixture in an 81% yield and 99.9% ee.

In this work, we demonstrate the utility of transition-metal catalysis as a powerful tool in organic synthesis, which enabled the concise and asymmetric synthesis of the active pharmaceutical ingredient ramelteon through a minimum number of synthetic steps from the monocyclic acetophenone precursor. We succeeded in developing only a six-step total asymmetric synthesis of ramelteon using iridium-catalyzed *O*-vinylation, Rh-catalyzed vinyl ether annulation via directed C–H bond activation, copper-catalyzed reduction of an α,β-unsaturated nitrile moiety under hydrosilylation conditions, and a nickel-catalyzed reduction nitrile group. Overall, the six-step synthesis provides ramelteon in 99.9% ee and 17% overall yield, which gives an average 74% yield per step. Our approach surpasses previously known approaches, which have applied a long 9–13 step total asymmetric synthesis of ramelteon or provided racemic ramelteon. Therefore, we believe that our report contributes important perspective on the high efficiency of transition-metal catalysis in the synthesis of difficult-to-make active pharmaceutical ingredients.

## Experimental Section

### General Information

Unless otherwise noted, all reactions were performed in dry round-bottom flasks. Unless otherwise stated, common reagents were obtained from a commercial source and used without further purification. Starting material 1-(3-hydroxyphenyl)ethanone **1** was purchased from Sigma-Aldrich. Dry solvents were used as purchased. Analytical thin-layer chromatography (TLC) was performed on Merck silica gel (60F_254_) plates (0.25 mm). Flash column chromatography was performed using a Biotage SP4 system. ^1^H NMR spectra were recorded at 500 MHz and ^13^C{^1^H} NMR spectra at 125 MHz on a Bruker Avance III 500 MHz spectrometer in CDCl_3_. ^1^H NMR chemical shifts are reported in parts per million (δ) relative to tetramethylsilane (TMS) with the TMS resonance employed as the internal standard (TMS, δ 0.00 ppm). When TMS was not present or clearly visible, the solvent resonance was employed as the internal standard (CDCl_3_, δ 7.26 ppm). Data are reported as follows: chemical shift, multiplicity (s = singlet, br s = broad singlet, d = doublet, t = triplet, q = quartet, m = multiplet), coupling constants (*J*, in hertz), and integration. ^13^C{^1^H} NMR chemical shifts are reported in ppm from the solvent resonance as the internal standard (CDCl_3_, δ 77.23 ppm). Chiral HPLC analysis of compound **8** obtained in HTS experiments was performed using the supercritical fluid chromatography method (for details see Supporting Information). Chiral HPLC analysis of compound **8** prepared in preparative experiments was performed on a Waters Alliance 2695 Separations module equipped with Waters Alliance 2487 Dual λ Absorbance Detector using the following: Chiralpak AD-H, 250 mm × 4.6 mm; flow rate, 1.0 mL/min; inj. volume, 10 μL; 30 °C; absorbance measurement at 230 nm; solvent, *n*-heptane/2-propanol = 90:10 (v/v); mobile phase, *n*-heptane/2-propanol = 95:5 (v/v). The elution times are as follows: (*S*)-**8**, 10.1 min; **7**, 12.0 min; (*R*)-**8**, 12.6 min (for details, see Supporting Information). Chiral HPLC analysis of ramelteon **9** was conducted on a Waters Alliance 2695 Separations module equipped with a Waters Alliance 2487 Dual λ Absorbance Detector using a method reported in literature.^37^ The elution times are (*S*)-**9**, 9.7 min and (*R*)-**9**, 14.0 min (for details, see Supporting Information). HRMS was recorded with an Agilent 6224 time-of-flight mass spectrometer equipped with a double orthogonal electrospray source at atmospheric pressure ionization (ESI) coupled to an HPLC instrument. DSC thermograms were acquired using the differential scanning calorimeter DSC 3^+^ Star^e^ System instrument (Mettler Toledo, Polaris Parkway Columbus, OH, USA) operating at 10 °C/min. FTIR spectra were collected with a Nicolet iS50FT-IR spectrometer (Thermo Fisher Scientific, Waltham, MA, USA).

#### Preparation of 1-(3-(Vinyloxy)phenyl)ethan-1-one (**3**)

1-(3-Hydroxyphenyl)ethanone (**1**) (5 g, 36.8 mmol) was suspended in dry toluene (37 mL), and dry sodium carbonate (2.34 g, 0.6 equiv) and [Ir(COD)Cl]_2_ (247 mg, 0.01 equiv) were added. Vinyl acetate (**2**) (6.8 mL, 2 equiv) was finally added, and the reaction was heated at 100 °C for 2 h using a thermostat system. The reaction was cooled down to room temperature, filtered, and concentrated under a vacuum. The residue was purified by flash chromatography (Biotage SNAP Cartridge KP-Sil using a EtOAc/*n*-heptane 2–11% gradient) to give 1-(3-(vinyloxy)phenyl)ethanone **3** (5.05 g, 85%) as a colorless oil: ^1^H NMR (500 MHz, CDCl_3_) δ 7.65 (dt, *J* = 7.6, 1.3 Hz, 1H), 7.59–7.54 (m, 1H), 7.40 (t, *J* = 7.9 Hz, 1H), 7.19 (ddd, *J* = 8.1, 2.6, 1.0 Hz, 1H), 6.66 (dd, *J* = 13.7, 6.1 Hz, 1H), 4.80 (dd, *J* = 13.7, 1.8 Hz, 1H), 4.50 (dd, *J* = 6.0, 1.8 Hz, 1H), 2.58 (s, 3H); ^13^C{^1^H} NMR (125 MHz, CDCl_3_) δ 197.3, 156.9, 147.5, 138.6, 129.8, 123.1, 121.9, 116.0, 96.1, 26.7; ν_max_ (neat)/cm^–1^ 3067, 3004, 2924, 1687, 1585, 1441, 1272, 1210, 956 cm^–1^; HRMS (ESI-TOF) *m*/*z* [M + H]^+^ calcd for C_10_H_11_O_2_ 163.0754, found 163.0748.

#### Preparation of 1-(2,3-Dihydrobenzofuran-4-yl)ethan-1-one (**4**)

1-(3-(Vinyloxy)phenyl)ethanone (**3**) (4.00 g, 24.7 mmol) was dissolved in dry toluene (242 mL), 4 Å molecular sieves (25 g, 1 g/mmol) and benzylamine (2.70 mL, 24.7 mmol) were added, and the reaction was heated at reflux for 18 h on an oil bath. The reaction was cooled down to room temperature, filtered, and concentrated. The residue was dissolved in toluene (20 mL), Ph_3_PRhCl (685 mg, 0.03 equiv) was added, and the reaction was heated for 18 h at 130 °C in a pressure reactor on an oil bath. The reaction was cooled down to room temperature, 1 N HCl (250 mL) was added, and the reaction was stirred for 2 h. Phases were separated, and the organic phase was washed successively with 1 N HCl (100 mL), water (100 mL), and brine (100 mL). Organic phase was dried over MgSO_4_ and filtered. Purification by filtration on a silica pad using dichloromethane (50 mL) afforded 1-(2,3-dihydrobenzofuran-4-yl)ethanone **4** (3.59 g, 90%) as an off-white solid: mp 48.4 °C (DSC onset) and 51.0 °C (DSC peak); ^1^H NMR (500 MHz, CDCl_3_) δ 7.34 (d, *J* = 0.9 Hz, 1H), 7.17 (t, *J* = 7.9 Hz, 1H), 6.93 (d, *J* = 7.9 Hz, 1H), 4.55 (t, *J* = 8.8 Hz, 2H), 3.50 (t, *J* = 8.8 Hz, 2H), 2.55 (s, 3H); ^13^C{^1^H} NMR (125 MHz, CDCl_3_) δ 198.9, 161.2, 133.9, 128.3, 128.0, 121.5, 113.5, 71.7, 31.1, 27.7; ν_max_ (KBr disc)/cm^–1^ 2982, 2965, 2907, 2893, 1678, 1585, 1454, 1268, 984, 943, 896 cm^–1^; HRMS (ESI-TOF) *m*/*z* [M + H]^+^ calcd for C_10_H_11_O_2_ 163.0754, found 163.0748.

#### Preparation of 1,2,6,7-Tetrahydro-8*H*-indeno[5,4-*b*]furan-8-one (**5**)

1-(2,3-Dihydrobenzofuran-4-yl)ethanone (**4**) (10 g, 61.7 mmol) was dissolved in dry dioxane (600 mL). *i-*Pr_2_NH × TFA (13.27 g, 1 equiv) and paraformaldehyde (3.7 g, 1 equiv) were added. The reaction was heated at reflux for 48 h on an oil bath. Additional portions of paraformaldehyde (3.70 g, 1 equiv) were added again after 6 and 24 h into the reaction. After, the reaction was partitioned between the brine/water mixture (1:1 v/v, 200 mL) and pentane (166 mL). The aqueous phase was re-extracted 3 times with pentane (110 mL). Combined pentane phases were washed with water and brine and dried over MgSO_4_. The solution was diluted to a total volume of 500 mL of pentane. This solution was added dropwise (0.5 mL/min) to a preheated 98% sulfuric acid solution (66 mL) at 40 °C under a nitrogen stream. At the end of addition, the reaction was cooled down to room temperature, and ice (116 mL) and *t*-BuOMe (116 mL) were added. The solution was stirred for 1 h and extracted 3 times with *t*-BuOMe/EtOAc (1:1 v/v, 150 mL). Combined organic phases were washed with water and 1 M NaHCO_3_ (170 mL), dried over MgSO_4_, and concentrated. Purification by flash chromatography (Biotage SNAP Cartridge KP-Sil using a EtOAc/*n*-heptane 2–40% gradient) furnished 1-(2,3-dihydrobenzofuran-4-yl)ethanone **4** (3.47 g, 35% recovered material) and pure 6,7-dihydro-1*H*-indeno[5,4-*b*]furan-8(2*H*)-one **5** (2.85 g, 27% yield and 41% yield based on consumed **4**) as an off white solid: mp 132.5 °C (DSC onset) and 133.5 °C (DSC peak); ^1^H NMR (500 MHz, CDCl_3_) δ 7.21 (d, *J* = 8.1 Hz, 1H), 7.02 (d, *J* = 8.2 Hz, 1H), 4.66 (t, *J* = 8.9 Hz, 2H), 3.48 (t, *J* = 8.9 Hz, 2H), 3.11–3.05 (m, 2H), 2.72–2.66 (m, 2H); ^13^C{^1^H} NMR (125 MHz, CDCl_3_) δ 207.4, 160.2, 147.1, 133.6, 125.6, 123.9, 115.6, 72.3, 37.1, 28.4, 25.4; ν_max_ (KBr disc)/cm^–1^ 2968, 2936, 1690, 1467, 1244, 939, 846 cm^–1^; HRMS (ESI-TOF) *m*/*z* [M + H]^+^ calcd for C_11_H_11_O_2_ 175.0754, found 175.0753.

#### Preparation of (*E*)-2-(1,2,6,7-Tetrahydro-8*H*-indeno[5,4-*b*]furan-8-ylidene)acetonitrile (**7**)

Compound **7** (1.56 g, yield 77%) as a white crystalline solid was prepared according to literature procedures:^3a−3c^ mp 140.5 °C (DSC onset) and 147.5 °C (DSC peak) (lit.^3a,3c^ mp = 149–151 °C and 146–151 °C); ^1^H NMR (500 MHz, CDCl_3_) δ 7.20 (d, *J* = 8.0 Hz, 1H), 7.01 (d, *J* = 8.0 Hz, 1H), 4.65 (t, *J* = 8.9 Hz, 2H), 3.47 (t, *J* = 8.9 Hz, 2H), 3.07 (t, *J* = 5.6 Hz, 2H), 2.72–2.65 (m, 2H); ^13^C{^1^H} NMR (125 MHz, CDCl_3_) δ 167.9, 160.3, 142.4, 135.1, 125.0, 122.0, 118.3, 113.1, 88.1, 71.6, 32.5, 29.5, 29.2; ν_max_ (KBr disc)/cm^–1^ 3083, 2978, 2966, 2915, 2206, 1602, 1479, 1441, 1242, 1142, 984 cm^–1^; HRMS (ESI-TOF) *m*/*z* [M + H]^+^ calcd for C_13_H_12_NO 198.0913, found 198.0917. ^1^H NMR data for compound **7** are in agreement with those from literature.^3a,3c^

#### Preparation of (*S*)-2-(1,6,7,8-Tetrahydro-2*H*-indeno[5,4-*b*]furan-8-yl)acetonitrile (**8**)

In a dry flask under a nitrogen atmosphere were added Walphos catalyst (**enant-L2**, 40 mg, 0.03 equiv) and copper acetate (7 mg, 0.03 equiv), followed by toluene (2.5 mL). The solution was cooled at 0 °C using a thermostat system. Polymethylhydrosiloxane (PMHS) (0.46 mL, 4 equiv) was added, and the reaction was stirred for 5–10 min. Compound **7** (250 mg, 1.27 mmol) was added, followed by *t*-BuOH (0.48 mL, 4 equiv). Dichloromethane was added (0.25 mL), and the reaction was slowly warmed up to room temperature. The reaction was stirred for 15 h. Then NaOH 1 N/10% NaCl solution (10 mL) was added, and the reaction mixture was stirred for 30 min. Phases were separated, and the aqueous solution was re-extracted twice with *t*-BuOMe. The combined organic phases were dried over MgSO_4_ and concentrated. Purification by flash chromatography (Biotage SNAP Cartridge KP-Sil using EtOAc/*n*-heptane 6–12% gradient) furnished compound (*S*)-**8** (0.18 g, 72% yield and 85% yield based on the consumed **7**, 97% ee) as an off-white solid and starting compound **7** (40 mg, 16% recovered material): mp 69.7 °C (DSC onset) and 72.0 °C (DSC peak); HPLC (Chiralpak AD-H, *n*-hepane/2-propanol = 95:5, flow rate = 1.0 mL/min, *l* = 230 nm) *t*_R_ = 10.1 min (major), *t*_R_ = 12.6 min (minor); ^1^H NMR (500 MHz, CDCl_3_) δ 6.99 (d, *J* = 8.0 Hz, 1H), 6.68 (d, *J* = 8.0 Hz, 1H), 4.63 (td, *J* = 9.4, 6.8 Hz, 1H), 4.58–4.51 (m, 1H), 3.50 (td, *J* = 8.2, 4.1 Hz, 1H), 3.30 (ddd, *J* = 15.2, 9.9, 8.0 Hz, 1H), 3.15 (ddd, *J* = 15.7, 9.8, 6.7 Hz, 1H), 2.98 (dt, *J* = 15.3, 7.6 Hz, 1H), 2.83 (ddd, *J* = 15.1, 8.6, 5.3 Hz, 1H), 2.69 (dd, *J* = 16.8, 5.3 Hz, 1H), 2.53 (dd, *J* = 16.8, 8.3 Hz, 1H), 2.49–2.38 (m, 1H), 2.01 (ddt, *J* = 13.2, 8.2, 5.1 Hz, 1H); ^13^C{^1^H} NMR (125 MHz, CDCl_3_) δ 159.7, 139.8, 135.7, 123.9, 122.3, 118.8, 108.6, 77.3, 77.0, 76.8, 71.2, 41.0, 32.3, 30.0, 28.4, 21.8; ν_max_ (neat)/cm^–1^ 2987, 2966, 2929, 2894, 2245, 1604, 1461, 1336, 1281, 1222, 1127, 1014, 989, 945 cm^–1^; HRMS (ESI-TOF) *m*/*z* [M + H]^+^ calcd for C_13_H_14_NO 200.1070, found 200.1073.

#### Preparation of Ramelteon (**9**)

Raney nickel in water (0.2 mL of homogenized suspension) was added to the reaction vessel and was washed by slurring/decantation with absolute EtOH (4 × 2 mL) and with dry THF (5 × 2 mL). Compound (*S*)-**8** (200 mg, 1 mmol) was dissolved in dry THF (11 mL) and was added to the activated nickel catalyst, followed by the addition of propionic anhydride (1.5 mL, 11.5 equiv). The reactor was sealed and flushed four times with a nitrogen atmosphere under a pressure of 0.5 MPa. Thereafter, the reactor was flushed four times with a hydrogen atmosphere under pressure of 0.4 MPa. The reaction mixture under 0.4 MPa of hydrogen was heated at 80 °C on a heating plate using internal thermometer control and stirred overnight. Then, the reactor was cooled down to room temperature and depressurized, and the reaction mixture was filtered on Celite. The obtained solution was diluted with toluene (20 mL) and 2 N NaOH (10 mL), and the reaction was stirred for 30 min. The phases were separated, and the organic phase was washed with 2 N NaOH (10 mL) and brine. The organic phase was dried over MgSO_4_ and concentrated. The solid was dissolved in EtOAc (2 mL), and hexane (20 mL) was slowly added to promote crystallization. The solid was filtered to give pure ramelteon **9** as a white crystalline solid (210 mg, 81%, 99.9% ee): mp 115.2 °C (DSC onset) and 116.0 °C (DSC peak); HPLC (Chiralpak AD-H, *n*-hexane/ethanol/methanesulfonic acid = 900:100:0.1, flow rate = 1.0 mL/min, *l* = 220 nm) *t*_R_ = 9.7 min (major), *t*_R_ = 14.0 min (minor);^37^^1^H NMR (500 MHz, CDCl_3_) δ 6.96 (d, *J* = 7.9 Hz, 1H), 6.62 (d, *J* = 8.0 Hz, 1H), 5.42 (s, 1H), 4.59 (ddd, *J* = 9.9, 8.6, 6.6 Hz, 1H), 4.52 (dt, *J* = 9.9, 8.3 Hz, 1H), 3.35 (dtd, *J* = 11.3, 6.1, 3.7 Hz, 2H), 3.25 (dt, *J* = 15.4, 9.1 Hz, 1H), 3.19 (dt, *J* = 8.9, 4.5 Hz, 1H), 3.11 (ddd, *J* = 15.7, 9.8, 6.6 Hz, 1H), 2.94–2.85 (m, 1H), 2.78 (ddd, *J* = 15.2, 8.6, 6.0 Hz, 1H), 2.34–2.23 (m, 1H), 2.18 (q, *J* = 7.6 Hz, 2H), 2.08–1.98 (m, 1H), 1.83 (ddt, *J* = 11.9, 8.4, 5.8 Hz, 1H), 1.70–1.59 (m, 3H), 1.15 (t, *J* = 7.6 Hz, 3H); ^13^C{^1^H} (125 MHz, CDCl_3_) δ 173.7, 159.4, 143.1, 135.8, 123.4, 122.2, 107.4, 77.3, 77.0, 76.8, 71.2, 42.2, 38.0, 33.5, 31.7, 30.7, 29.8, 28.6, 9.9; ν_max_ (neat)/cm^–1^ 3313, 2971, 2934, 2861, 1641, 1544, 1462, 1232, 1210, 991, 810 cm^–1^; HRMS (ESI-TOF) *m*/*z* [M + H]^+^ calcd for C_16_H_22_NO_2_ 260.1645, found 260.1649.

## Supporting Information Available

Expanded discussion on the previous synthetic strategies towards ramelteon, additional experimental details on the screening of the asymmetric reduction step, copies of NMR spectra, IR spectra, and DSC thermograms of prepared compounds and HPLC chromatograms for the determination of chiral purity (PDF)

## Dedication

This manuscript is dedicated in memoriam to Emeritus Professor Dr. Miha Tišler (1926-2021), University of Ljubljana, Slovenia.
