# Supplementary material for: Concise Six-Step Asymmetric Approach to Ramelteon from an Acetophenone Derivative Using Ir, Rh, Cu, and Ni Catalysis
Source: J Org Chem. 2021 Sep 30;87(4):2129–35. doi: 10.1021/acs.joc.1c01614 (PMC8859824; doi:10.1021/acs.joc.1c01614)
Supplement: Supplementary file 1 — jo1c01614_si_001.pdf [file jo1c01614_si_001.pdf]

Supporting Information

Concise Six-Step Asymmetric Approach to Ramelteon from Acetophenone Derivative Using Ir, Rh, Cu and Ni Catalysis

Jerome Cluzeau,[a*] Ulrike Nettekoven,[b] Miroslav Planinc Kovačevič[a] and Zdenko Časar[a,c*]

[a] Lek Pharmaceuticals d.d., Sandoz Development Center Slovenia, Kolodvorska 27, 1234 Mengeš, Slovenia

[b] Solvias AG, Römerpark 2, 4303 Kaiseraugst, Switzerland

[c] University of Ljubljana, Faculty of Pharmacy, Aškerčeva c. 7, SI-1000 Ljubljana, Slovenia

** E-mail: jerome.cluzeau@sandoz.com*

** E-mail: zdenko.casar@sandoz.com, zdenko.casar@ffa.uni-lj.si*

Contents of Supporting Information:

1. Expanded discussion on the previous synthetic strategies towards ramelteon S-2
2. Enantioselective CuH Reduction of (7) – High-Throughput Screening S-4
Figure S1. List of the tested ligands S-5
Figure S2. List of the tested ligands S-6
Table S1. Plate conditions and hydrogenation results S-7
3. Schemes of reactions described in the experimental section of the manuscript S-9
4. Copies of ^{1}H NMR spectra, ^{13}C{^{1}H} NMR spectra, IR spectra, DSC thermograms and HPLC chromatograms S-11
^{1}H NMR of 1-(3-(vinyloxy)phenyl)ethan-1-one (**3**) S-11
^{13}C{^{1}H} NMR of 1-(3-(vinyloxy)phenyl)ethan-1-one (**3**) S-12
IR spectra of 1-(3-(vinyloxy)phenyl)ethan-1-one (**3**) S-13
^{1}H NMR of 1-(2,3-dihydrobenzofuran-4-yl)ethan-1-one (**4**) S-14
^{13}C{^{1}H} NMR of 1-(2,3-dihydrobenzofuran-4-yl)ethan-1-one (**4**) S-15
IR spectra of 1-(2,3-dihydrobenzofuran-4-yl)ethan-1-one (**4**) S-16
DSC thermogram of 1-(2,3-dihydrobenzofuran-4-yl)ethan-1-one (**4**) S-17
^{1}H NMR of 1,2,6,7-tetrahydro-8*H*-indeno[5,4-*b*]furan-8-one (**5**) S-18
^{13}C{^{1}H} NMR of 1,2,6,7-tetrahydro-8*H*-indeno[5,4-*b*]furan-8-one (**5**) S-19
IR spectra of 1,2,6,7-tetrahydro-8*H*-indeno[5,4-*b*]furan-8-one (**5**) S-20
DSC thermogram of 1,2,6,7-tetrahydro-8*H*-indeno[5,4-*b*]furan-8-one (**5**) S-21
^{1}H NMR of (*E*)-2-(1,2,6,7-tetrahydro-8*H*-indeno[5,4-*b*]furan-8-ylidene)acetonitrile (**7**) S-22
^{13}C{^{1}H} NMR of (*E*)-2-(1,2,6,7-tetrahydro-8*H*-indeno[5,4-*b*]furan-8-ylidene)acetonitrile (**7**) S-23
IR spectra of (*E*)-2-(1,2,6,7-tetrahydro-8*H*-indeno[5,4-*b*]furan-8-ylidene)acetonitrile (**7**) S-24
DSC thermogram of (*E*)-2-(1,2,6,7-tetrahydro-8*H*-indeno[5,4-*b*]furan-8-ylidene)acetonitrile (**7**) S-25
^{1}H NMR of (*S*)-2-(1,6,7,8-tetrahydro-2*H*-indeno[5,4-*b*]furan-8-yl)acetonitrile (**8**) S-26
^{13}C{^{1}H} NMR of (*S*)-2-(1,6,7,8-tetrahydro-2*H*-indeno[5,4-*b*]furan-8-yl)acetonitrile (**8**) S-27
IR spectra of (*S*)-2-(1,6,7,8-tetrahydro-2*H*-indeno[5,4-*b*]furan-8-yl)acetonitrile (**8**) S-28
DSC thermogram of (*S*)-2-(1,6,7,8-tetrahydro-2*H*-indeno[5,4-*b*]furan-8-yl)acetonitrile (**8**) S-29
^{1}H NMR of ramelteon (**9**) S-30
^{13}C{^{1}H} NMR of ramelteon (**9**) S-31
IR spectra of ramelteon (**9**) S-32
DSC thermogram of ramelteon (**9**) S-33
Chiral HPLC of (*S*)-2-(1,6,7,8-tetrahydro-2*H*-indeno[5,4-*b*]furan-8-yl)acetonitrile (**8**) S-34
Chiral HPLC of ramelteon (**9**) S-35

1. *Expanded discussion on the previous synthetic strategies towards ramelteon*

One of the primary synthetic routes started from 2,3-dihydrobenzofuran, which was converted to racemic ramelteon in 11 steps via 4,5-dibromo-1,2,6,7-tetrahydro-8*H*-indeno[5,4-*b*]furan-8-one intermediate (Scheme S1, entry 1).[3] In another primary synthetic route chiral ramelteon was obtained in 13 steps from the starting 6-methoxy-2,3-dihydro-1*H*-inden-1-one *via* (*S*)-*N*-(2-(5-bromo-6-hydroxy-7-(hydroxymethyl)-2,3-dihydro-1*H*-inden-1-yl)ethyl)propionamide (Scheme S1 entry 2).[3] An asymmetric synthesis of ramelteon starting from 4-bromo-2,3-dihydrobenzofuran was accomplished in 9 steps using asymmetric Michael addition in the key step (Scheme S1, entry 3).[4] The only known synthetic route that started from monocyclic precursor provided ramelteon in 13 steps and used intramolecular asymmetric Michael reaction of tert-butanesulfinyl ketimines for the diastereoselective synthesis of indanone core (Scheme S1, entry 4).[5] Zhou *et al*. reported 4 step synthesis of ramelteon starting from tricyclic 4,5-dibromo-1,2,6,7-tetrahydro-8*H*-indeno[5,4-*b*]furan-8-one derivative, that must be prepared in 7 steps from 2,3-dihydrobenzofuran.[3] In the key step racemic 2-(1,6,7,8-tetrahydro-2*H*-indeno[5,4-*b*]furan-8-yl)ethan-1-amine was formed and its optical purity was subsequently upgraded to 98.6% *ee via* resolution crystallization using dibenzoyl-*L*-tartaric acid (Scheme S1, entry 5).[6] Recently, Zhou *et al*. reported a concise 3 step synthesis of racemic ramelteon in 26% yield starting from advanced bicyclic 2,3-dihydrobenzofuran-4-amine starting material. In the key step the intermolecular Catellani-type alkylation and intramolecular redox-relay Heck cyclization cascade was applied for the assembly of key 2-(1,6,7,8-tetrahydro-2*H*-indeno[5,4-b]furan-8-yl)acetaldehyde intermediate.[7] Nevertheless, 2,3-dihydrobenzofuran-4-amine precursor must be prepared from 3-methoxyaniline in additional 3 steps in 16-21% yield according to the literature procedures (Scheme S1, entry 6).[11]

List of references in order as presented in the manuscript:

(3) (a) Uchikawa, O.; Fukatsu, K.; Tokunoh, R.; Kawada, M.; Matsumoto, K.; Imai, Y.; Hinuma, S.; Kato, K.; Nishikawa, H.; Hirai, K.; Miyamoto, M.; Ohkawa, S. Synthesis of a Novel Series of Tricyclic Indan Derivatives as Melatonin Receptor Agonists. *J. Med. Chem.* **2002**, *45*, 4222–4239. (b) Ohkawa, S.; Uchikawa, O.; Fukatsu, K.; Miyamoto, M. Tricyclic compounds, their production and use. US 6034239, March 7, 2000. (c) Yamano, T.; Yamashita, M.; Adachi, M.; Tanaka, M.; Matsumoto, K.; Kawada, M.; Uchikawa, O.; Fukatsu, K.; Ohkawa, S. Approach to the Stereoselective Synthesis of Melatonin Receptor Agonist Ramelteon via Asymmetric Hydrogenation. *Tetrahedron: Asymmetry* **2006**, *17*, 184–190. (d) Sakya, S. M.; Li, J., Liu, K. K.-C. Synthetic Approaches to the 2005 New Drugs, *Mini-Rev. Med. Chem*. **2007**, *7*, 429–450. (e) Yamashita, M.; Yamano, T. Synthesis of Melatonin Receptor Agonist Ramelteon via Rh-catalyzed Asymmetric Hydrogenation of an Allylamine. *Chem. Lett*. **2009**, *38*, 100–101.

(4) Zhang, X.; Yuan, W.; Luo, Y.; Huang, Q.-Q.; Lu, W. Stereoselective Synthesis of Melatonin Receptor Agonist Ramelteon via Asymmetric Michael Addition. *Heterocycles* **2012**, *85*, 73–84.

(5) Fu, X. D.; Guo, X. Q.; Li, X.W.; He, L. D.; Yang, Y. S.; Chen, Y. X. Synthesis of the Melatonin Receptor Agonist Ramelteon Using a Tandem C–H Activation–Alkylation/Heck Reaction and Subsequent Asymmetric Michael Addition. *Tetrahedron: Asymmetry* **2013**, *24*, 827–832.

(6) Xiao, S.; Chen, C.; Li, H.; Lin, K.; Zhou, W. A Novel and Practical Synthesis of Ramelteon. *Org. Process Res. Dev*. **2015**, *19*, 373−377.

(7) Gao, S.; Qian, G.; Tang, H.; Yang, Z.; Zhou, Q. Three-Step Total Synthesis of Ramelteon via a Catellani Strategy. *ChemCatChem* **2019**, *11*, 5762−5765.

(8) Wang, B.; Zhang, L.; Fu, K.; Luo, Y.; Lu, W.; Tang, J. An Efficient Synthesis of 1,2,6,7-Tetrahydro-8*H*-indeno[5,4-*b*]furan-8-one. *Org. Prep. Proced. Int*. **2009**, *41*, 309–314,

(9) Yu, S. B.; Liu, H. M.; Luo, Y.; Lu, W. Synthesis of the Key Intermediate of Ramelteon. *Chin. Chem. Lett.* **2011**, *22*, 264–267.

(10) Zhixiong, H.; Chenglong, W.; Zhipeia, S.; Yong, D. An Improved Synthesis of 1,2,6,7-Tetrahydro-8*H*-indeno[5,4-b]furan-8-one. *Chin. J. Org. Chem.* **2012**, *32*, 2368–2372.

(11) (a) Favor, D. A.; Johnson, D. S.; Powers, J. J.; Li, T.; Madabattula, R. Synthesis of Chromanyl and Dihydrobenzofuranyl Piperazines. *Tetrahedron Lett.* **2007**, *48*, 3039–3041. (b) Hagiwara, M.; Hosoya, T.; Kii, I.; Onogi, H.; Compound and pharmaceutical composition for neuropsychological disorder or malignant tumor. EP EP2881397 A1, June 10, 2015. (c) Roffey, J. R. A.; Davidson, J. E. P.; Mansell, H. L.; Hamlyn, R. J.; Adams, D. R. Condensed indoline derivatives and their use as 5HT, in particular 5HT2C, receptor ligands. US 2005187282 A1, August 25, 2005.

Entry 1: 11 steps from bicyclic precursor, racemic (ref. 3)

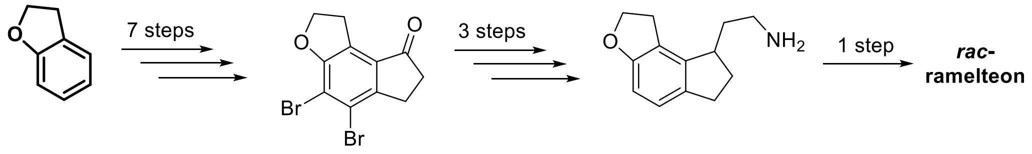

Entry 2: 13 steps from bicyclic precursor, asymmetric (ref. 3)

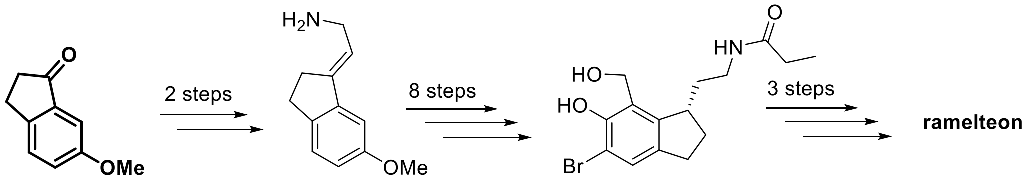

Entry 3: 9 steps from bicyclic precursor, asymmetric (ref. 4)

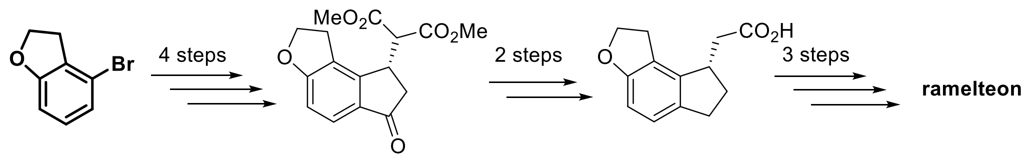

Entry 4: 13 steps from monocyclic precursor, asymmetric (ref. 5)

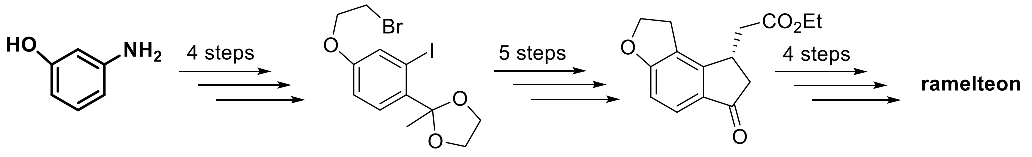

Entry 5: 4 steps from tricyclic precursor, racemic + resolution crystallization (ref. 6)

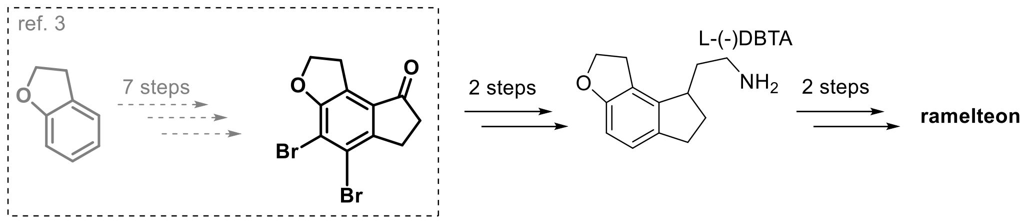

Entry 6: 3 steps from advanced bicyclic precursor, racemic (ref. 7)

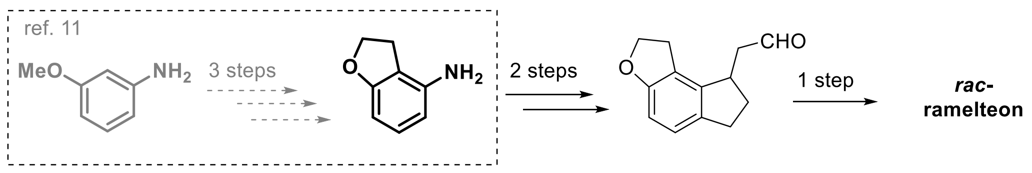

Scheme S1. Previously known synthetic strategies to ramelteon.

2. *Enantioselective CuH Reduction of (7) – High-Throughput Screening*

Reaction scheme:

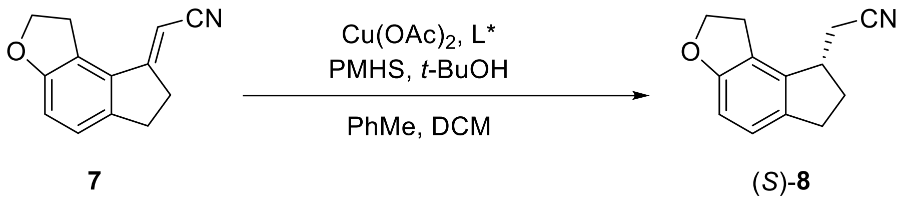

Scheme S2. Enantioselective CuH Reduction of **7**.

The HT screening of the desired transformation was conducted using in total:

• 48 different ligands (complete list of the tested ligands is shown in **Figure S1** and **Figure S2**)
• 1 metal precursor: $Cu(OAc)_2$
• Toluene and DCE as solvent
• S/C ratios of 25 and 100

in 96 experiments on the reaction plate. Conditions were chosen to be room temperature, argon atmosphere and 20 h of reaction time.

The following aspects of the plate results are noteworthy:

• Several ligand hits were identified giving high enantioselecitivity, conversion and chemoselectivity.
• The variation in the S/C ratio allows for a good judgement of catalyst productivity – not all hits gave full conversion also at the higher S/C ratio of 100.

Results of the screening are presented in **Table S1**.

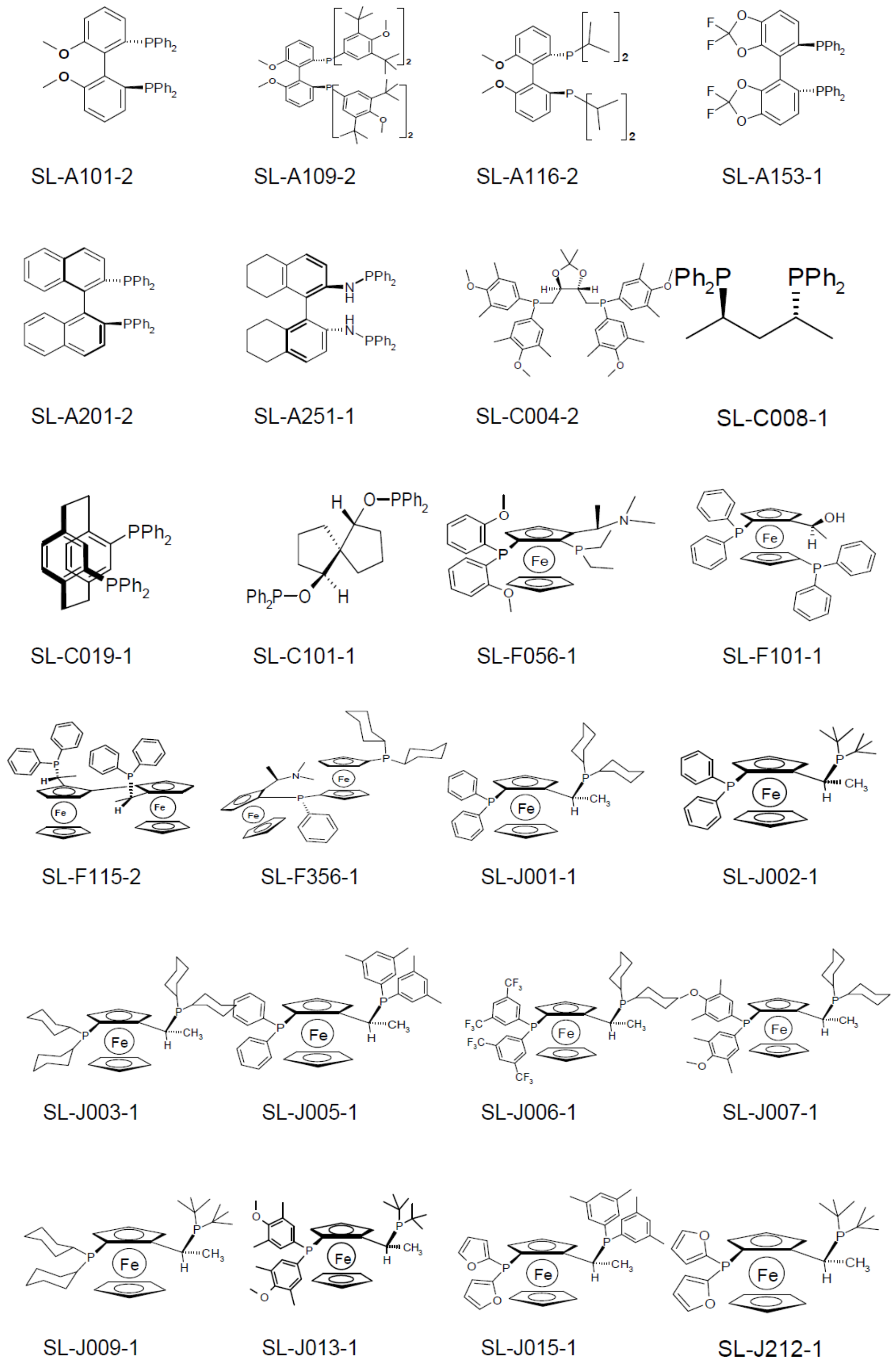

Figure S1. List of the tested ligands.

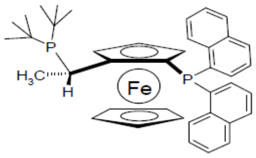

SL-J216-2

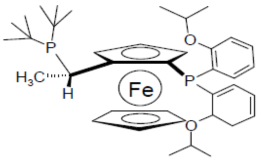

SL-J226-2

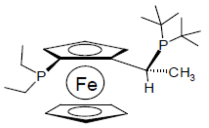

SL-J301-1

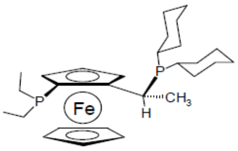

SL-J302-1

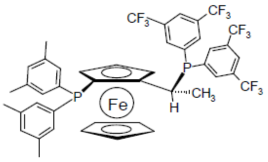

SL-J412-1

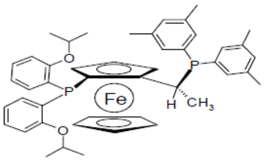

SL-J431-1

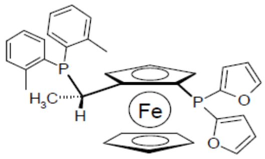

SL-J452-2

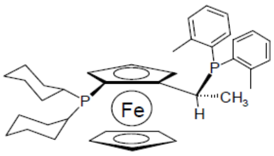

SL-J504-1

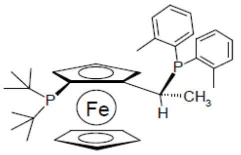

SL-J505-1

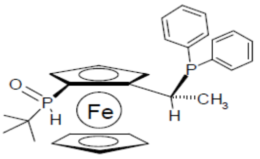

SL-J681-1

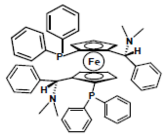

SL-M001-1

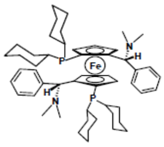

SL-M002-1

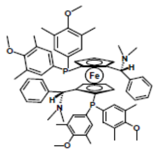

SL-M004-1

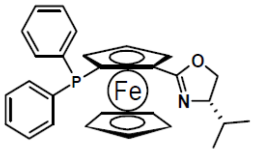

SL-N003-2

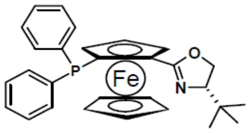

SL-N004-2

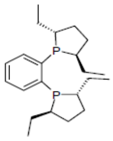

SL-P102-1

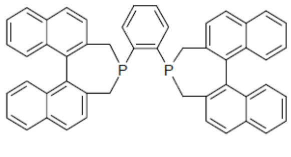

SL-P111-2

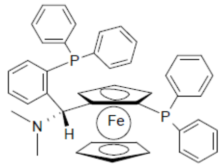

SL-T001-1

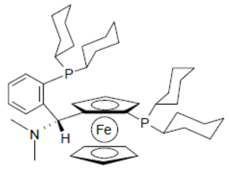

SL-T002-1

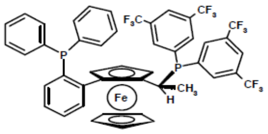

SL-W001-1

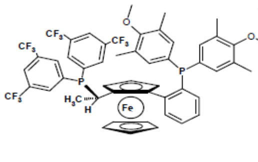

SL-W005-2

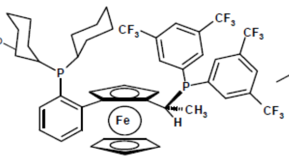

SL-W008-1

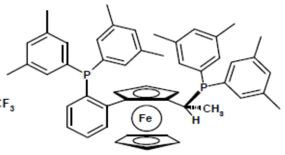

SL-W009-1

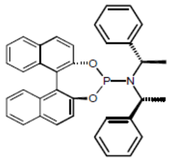

SL-Z102-1

Figure S2. List of the tested ligands.

Table S1. Plate conditions and hydrogenation results.

Row	Col	Ligand	S/C	Product 1 (S) Area% (SFC)	Product 2 (R) Area% (SFC)	SM Area% (SFC)	BP 6.0' Area% (SFC)	Conversion (SFC)	%e.e. (SFC)	Selectivity (SFC)
A	1	SL-J001-1	25.0	10.0	90.0			100.0	-79.9	100.0
A	2	SL-J001-1	100.0	8.9	91.1			100.0	-82.1	100.0
A	3	SL-J226-2	25.0	4.6	2.0	93.4		n.d.	n.d.	n.d.
A	4	SL-J226-2	100.0	2.8	0.9	87.9	8.1	12.1	53.6	30.3
A	5	SL-J007-1	25.0	11.1	88.9			100.0	-77.8	100.0
A	6	SL-J007-1	100.0	11.9	87.3	0.5		99.5	-76.0	99.8
A	7	SL-W005-2	25.0	1.5	88.2			100.0	-96.6	89.7
A	8	SL-W005-2	100.0	2.2	96.8			100.0	-95.7	99.0
A	9	SL-A109-2	25.0	51.0	34.4	12.4	1.0	87.6	19.4	97.5
A	10	SL-A109-2	100.0	34.8	22.9	39.0	3.3	61.0	20.6	94.5
A	11	SL-F101-1	25.0	30.0	17.7	52.4		47.6	25.8	100.0
A	12	SL-F101-1	100.0	14.0	9.3	70.5	6.0	29.5	20.2	79.0
B	1	SL-J002-1	25.0	8.1	13.3	78.6		21.4	-24.6	100.0
B	2	SL-J002-1	100.0	6.1	8.5	85.3		14.7	-16.4	100.0
B	3	SL-J301-1	25.0	31.3	4.8	59.6	4.3	40.4	73.5	89.4
B	4	SL-J301-1	100.0	40.5	6.9	52.5		47.5	70.8	100.0
B	5	SL-J302-1	25.0	55.8	42.9	1.3		98.7	13.0	100.0
B	6	SL-J302-1	100.0	31.2	21.6	43.2	4.0	56.8	18.2	93.0
B	7	SL-W008-1	25.0	1.4	0.8	85.6		14.4	30.0	15.4
B	8	SL-W008-1	100.0	0.7	0.7	97.1		n.d.	n.d.	n.d.
B	9	SL-A116-2	25.0	2.0	9.8	80.9	7.3	19.1	-66.7	61.6
B	10	SL-A116-2	100.0	0.4	0.8	89.6	8.7	10.4	-35.2	11.3
B	11	SL-Z102-1	25.0	1.9	0.8	97.3		n.d.	n.d.	n.d.
B	12	SL-Z102-1	100.0			92.5	7.5	n.d.	n.d.	n.d.
C	1	SL-J003-1	25.0	12.2	31.8	56.1		43.9	-44.6	100.0
C	2	SL-J003-1	100.0	16.4	42.5	41.1		58.9	-44.4	100.0
C	3	SL-J006-1	25.0	27.4	72.6			100.0	-45.2	100.0
C	4	SL-J006-1	100.0	25.7	74.3			100.0	-48.5	100.0
C	5	SL-M001-1	25.0	9.6	2.8	87.6		12.4	54.9	100.0
C	6	SL-M001-1	100.0	8.3	2.7	81.3	7.3	18.7	50.8	58.7
C	7	SL-A201-2	25.0	28.0	63.0	9.0		91.0	-38.4	100.0
C	8	SL-A201-2	100.0	24.6	51.9	23.5		76.5	-35.8	100.0
C	9	SL-A153-1	25.0	53.6	34.7	11.7		88.3	21.4	100.0
C	10	SL-A153-1	100.0	54.4	38.2	6.3		93.7	17.5	98.8
C	11	SL-P111-2	25.0	61.4	38.0	0.7		99.3	23.5	100.0
C	12	SL-P111-2	100.0	24.8	17.3	53.3	4.6	46.7	17.7	90.2
D	1	SL-J005-1	25.0	47.5	51.8	0.7		99.3	-4.3	100.0
D	2	SL-J005-1	100.0	47.3	52.7			100.0	-5.3	100.0
D	3	SL-J412-1	25.0	12.2	86.5			100.0	-75.3	98.7
D	4	SL-J412-1	100.0	14.2	85.3			100.0	-71.4	99.5
D	5	SL-M002-1	25.0	4.0	2.6	93.3		n.d.	n.d.	n.d.
D	6	SL-M002-1	100.0	1.3	2.0	96.0		n.d.	n.d.	n.d.
D	7	SL-A101-2	25.0	46.5	43.8	9.7		90.3	2.9	100.0
D	8	SL-A101-2	100.0	27.7	28.5	43.8		56.2	-1.4	100.0
D	9	SL-A251-1	25.0	5.2	3.6	90.0		10.0	17.7	88.0
D	10	SL-A251-1	100.0	3.4	2.4	86.2	7.7	13.8	17.4	42.1
D	11	SL-P102-1	25.0	32.2	64.7	3.1		96.9	-33.5	100.0
D	12	SL-P102-1	100.0	35.2	63.9	0.7		99.3	-28.9	99.8
E	1	SL-J009-1	25.0	3.4	3.9	92.7		n.d.	n.d.	n.d.
E	2	SL-J009-1	100.0	4.3	6.6	89.1		10.9	-21.6	100.0
E	3	SL-J431-1	25.0	85.4	14.6			100.0	70.7	100.0
E	4	SL-J431-1	100.0	82.6	16.0	1.3		98.7	67.5	100.0
E	5	SL-M004-1	25.0	93.0	7.0			100.0	86.0	100.0
E	6	SL-M004-1	100.0	84.0	5.5	10.5		89.5	87.8	100.0
E	7	SL-N003-2	25.0	2.2	15.8	75.5	6.6	24.5	-75.6	73.2
E	8	SL-N003-2	100.0	1.4	7.9	82.9	7.2	17.1	-69.7	54.7
E	9	SL-C019-1	25.0	2.2	1.3	61.1	5.3	38.9	n.d.	8.9
E	10	SL-C019-1	100.0	0.6	19.0	75.1		24.9	-93.4	78.7
E	11	SL-W009-1	25.0	95.3	4.7			100.0	90.6	100.0
E	12	SL-W009-1	100.0	92.2	6.6	0.9		99.1	86.6	99.7
F	1	SL-J013-1	25.0	12.2	37.6	50.2		49.8	-51.0	100.0

F	2	SL-J013-1	100.0	14.8	43.0	42.2		57.8	-48.8	100.0
F	3	SL-J504-1	25.0	13.7	21.1	65.2		34.8	-21.1	100.0
F	4	SL-J504-1	100.0	10.4	13.6	76.0		24.0	-13.5	100.0
F	5	SL-T001-1	25.0	12.2	34.3	49.3	4.2	50.7	-47.6	91.7
F	6	SL-T001-1	100.0	2.2	35.4	62.4		37.6	-88.2	100.0
F	7	SL-F356-1	25.0	1.4	0.4	98.3		n.d.	n.d.	n.d.
F	8	SL-F356-1	100.0	1.2	0.6	98.2		n.d.	n.d.	n.d.
F	9	SL-C101-1	25.0	20.3	2.9	76.8		23.2	74.9	100.0
F	10	SL-C101-1	100.0	21.1	3.1	75.9		24.1	74.6	100.0
F	11	SL-N004-2	25.0	2.6	7.6	82.8	7.0	17.2	-49.2	59.2
F	12	SL-N004-2	100.0		2.2	95.9		n.d.	n.d.	n.d.
G	1	SL-J015-1	25.0	19.3	46.5	34.2		65.8	-41.3	100.0
G	2	SL-J015-1	100.0	29.7	69.9	0.4		99.6	-40.4	100.0
G	3	SL-J505-1	25.0	2.1	6.1	91.8		n.d.	n.d.	n.d.
G	4	SL-J505-1	100.0		0.8	97.8		n.d.	n.d.	n.d.
G	5	SL-T002-1	25.0	7.5	2.7	89.7		10.3	46.5	100.0
G	6	SL-T002-1	100.0	16.5	5.0	78.3		21.7	53.7	98.8
G	7	SL-C004-2	25.0	34.9	65.1			100.0	-30.3	100.0
G	8	SL-C004-2	100.0	35.0	65.0			100.0	-30.1	100.0
G	9	SL-F115-2	25.0	3.4	4.4	92.2		n.d.	n.d.	n.d.
G	10	SL-F115-2	100.0	3.2	9.3	80.8	6.8	19.2	-49.2	64.9
G	11	SL-J216-2	25.0	2.2	7.5	90.2		n.d.	n.d.	n.d.
G	12	SL-J216-2	100.0	1.8	2.0	88.4	7.8	11.6	-4.1	32.6
H	1	SL-J212-1	25.0	13.0	53.0	34.1		65.9	-60.6	100.0
H	2	SL-J212-1	100.0	11.4	51.5	35.8		64.2	-63.7	98.0
H	3	SL-J681-1	25.0	28.4	40.9	30.7		69.3	-18.1	100.0
H	4	SL-J681-1	100.0	19.2	26.4	54.4		45.6	-16.0	100.0
H	5	SL-W001-1	25.0	77.7	1.6	13.4		86.6	96.0	91.6
H	6	SL-W001-1	100.0	40.7	0.5	58.5		41.5	97.5	99.3
H	7	SL-C008-1	25.0	17.4	82.2	0.4		99.6	-65.1	100.0
H	8	SL-C008-1	100.0	19.1	80.3	0.3		99.7	-61.6	99.7
H	9	SL-F056-1	25.0	31.4	68.3	0.3		99.7	-36.9	100.0
H	10	SL-F056-1	100.0	34.1	65.1	0.5		99.5	-31.2	99.7
H	11	SL-J452-2	25.0	7.2	4.3	81.0	7.3	19.0	25.2	60.4
H	12	SL-J452-2	100.0	3.1	2.7	94.0		n.d.	n.d.	n.d.

Reaction Conditions: 83 μmol of substrate (**7**), $Cu(OAc)_2$ as a metal precursor, PMHS:*t*-BuOH:PhMe:DCE = 52:40:240:10 μL. Argon atmosphere, room temperature 20 h. Conversion, yield and enantiomeric excess were determined by Supercritical Fluid Chromatography. n.d.: not determined.

Supercritical Fluid Chromatography method conditions

Apparatus Supercritical Fluid Chromatography, Berger Instruments

Column: Chirapak AD-H, 4.6 × 250 mm

Eluents: CO_2, 2-propanol

Flow: 1.5 mL/min

Outlet pressure: 100 bar

Temperature: 40 °C

Gradient: 20% (2-propanol), hold 6 min, 60%/min to 40%, hold 2 min, 60%/min to 20%

Detector, Wavelength: UV, 210 nm

Injection volume: 5 μL Loop

Acquisition time approx. 9 minutes

Sample preparation: 1- 3 mg in 1000 μL MeOH

Retention times:

Starting material **7**: 5.7 min

Product (*S*)-**8**: 3.9 min

Product (*R*)-**8**: 4.6 min

3. Schemes of reactions described in the experimental section of the manuscript

Scheme S3. Preparation of 1-(3-(vinyloxy)phenyl)ethan-1-one (3)

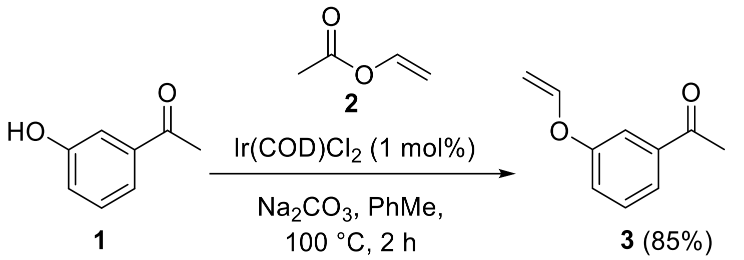

Scheme S4. Preparation of 1-(2,3-dihydrobenzofuran-4-yl)ethan-1-one (4)

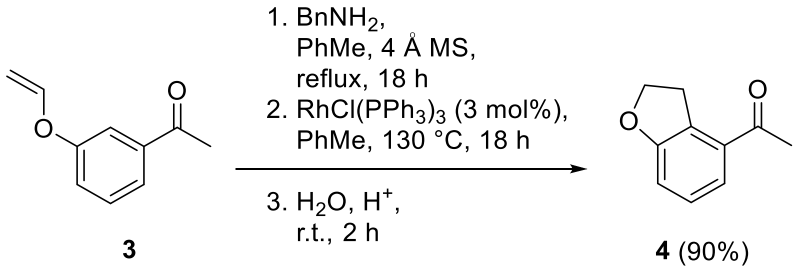

Scheme S5. Preparation of 1,2,6,7-tetrahydro-8*H*-indeno[5,4-*b*]furan-8-one (5)

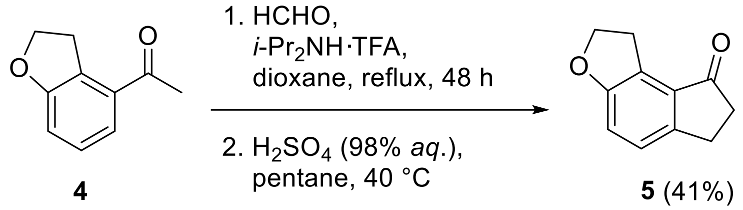

Scheme S6. Preparation of (*E*)-2-(1,2,6,7-tetrahydro-8*H*-indeno[5,4-*b*]furan-8-ylidene)acetonitrile (7)

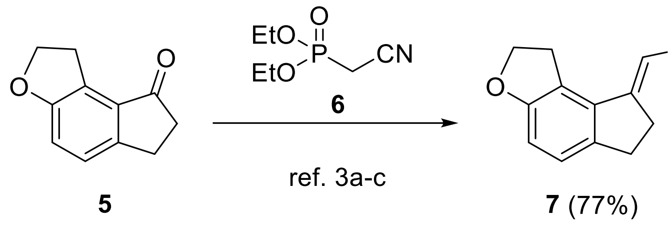

Scheme S7. Preparation of (*S*)-2-(1,6,7,8-tetrahydro-2*H*-indeno[5,4-*b*]furan-8-yl)acetonitrile (8)

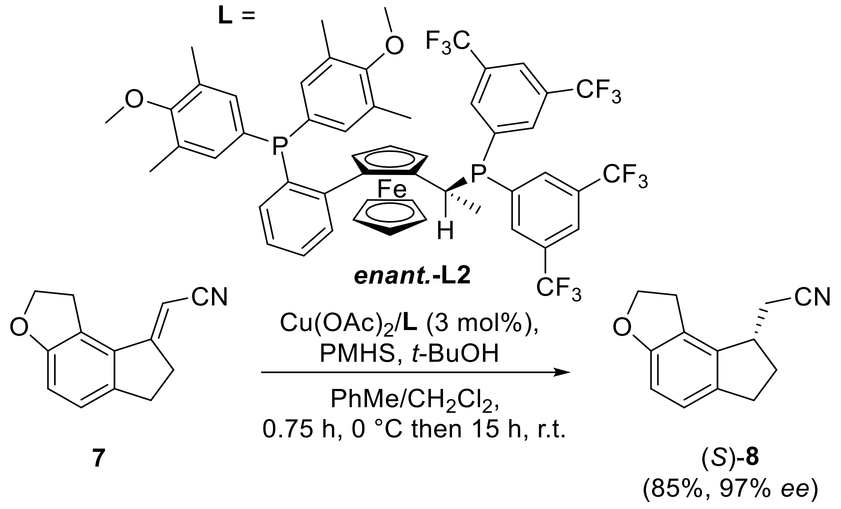

Scheme S8. Preparation of ramelteon (9)

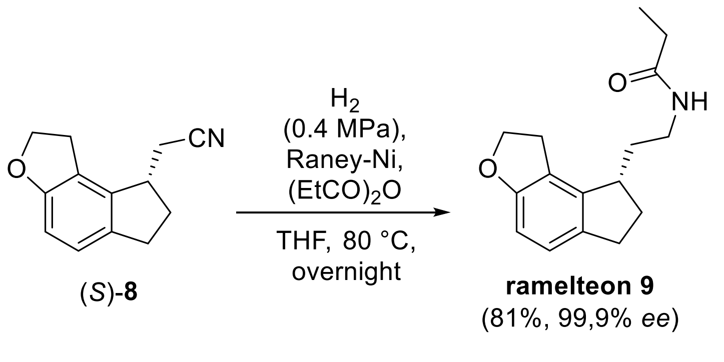

4. Copies of ^{1}H NMR spectra, ^{13}C{^{1}H} NMR spectra, IR spectra, DSC thermograms and HPLC chromatograms

^{1}H NMR (500 MHz, Chloroform-*d*) of 1-(3-(vinyloxy)phenyl)ethan-1-one (**3**)

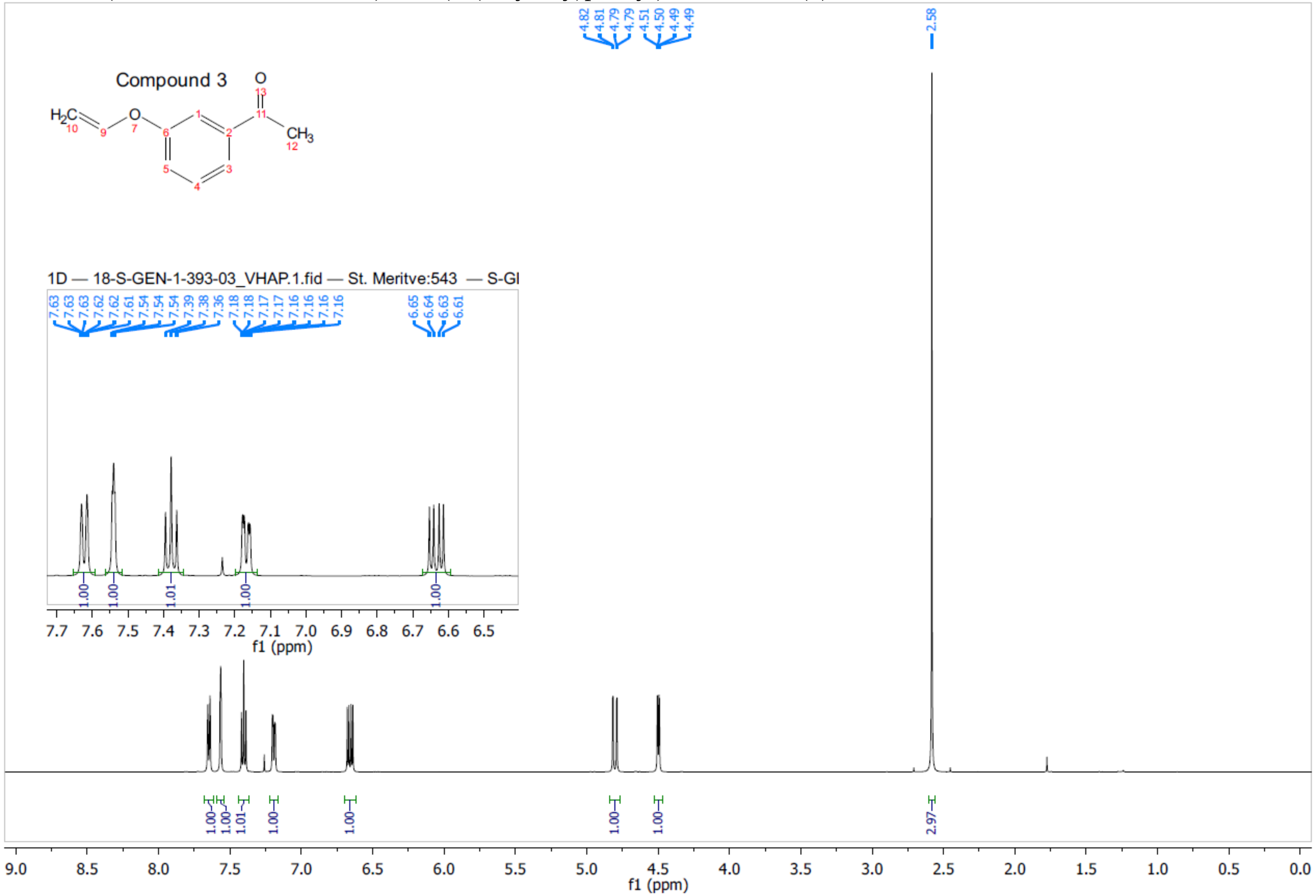

$^{13}C\{^1H\}$ NMR (125 MHz, Chloroform-*d*) of 1-(3-(vinyloxy)phenyl)ethan-1-one (**3**)

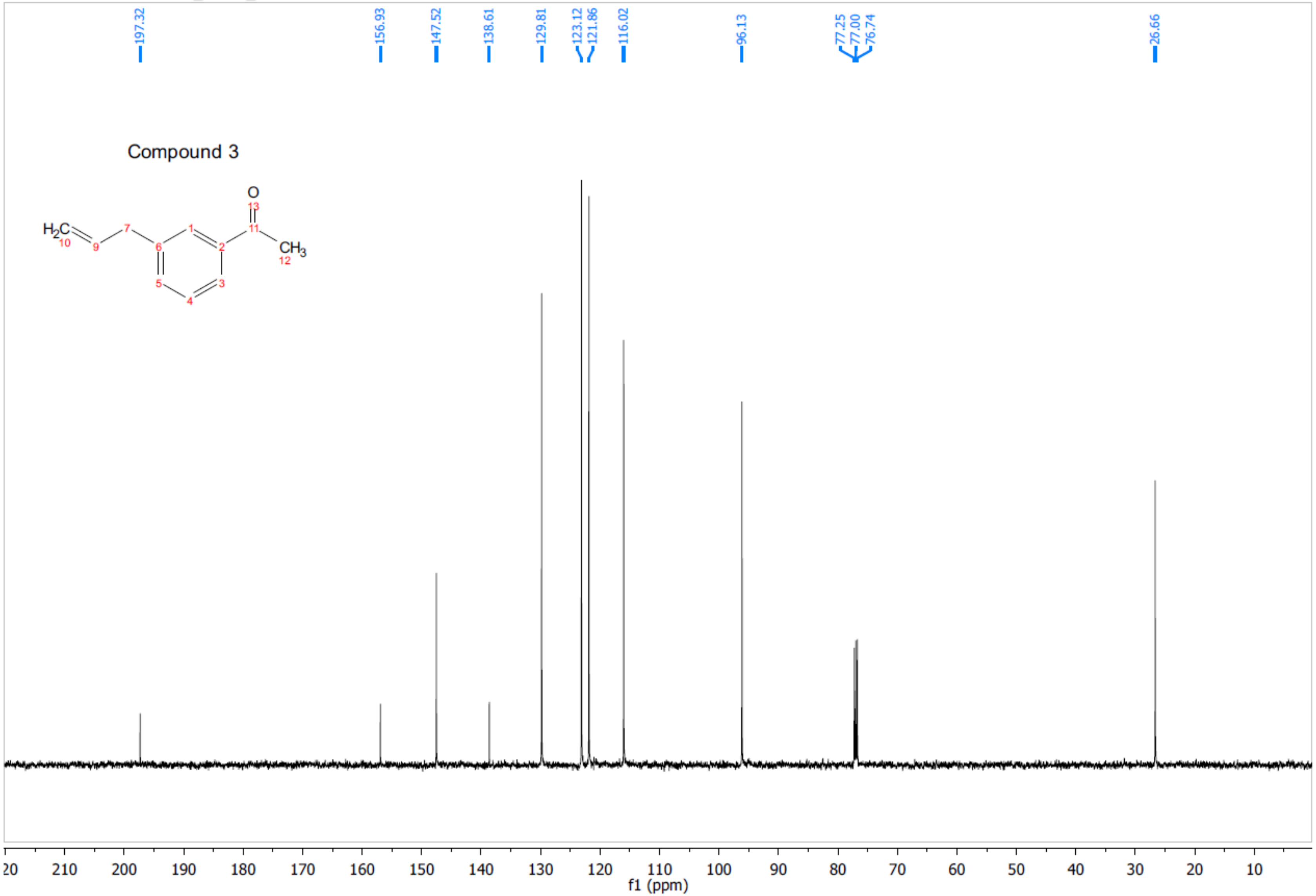

IR spectra of 1-(3-(vinyloxy)phenyl)ethan-1-one (**3**)

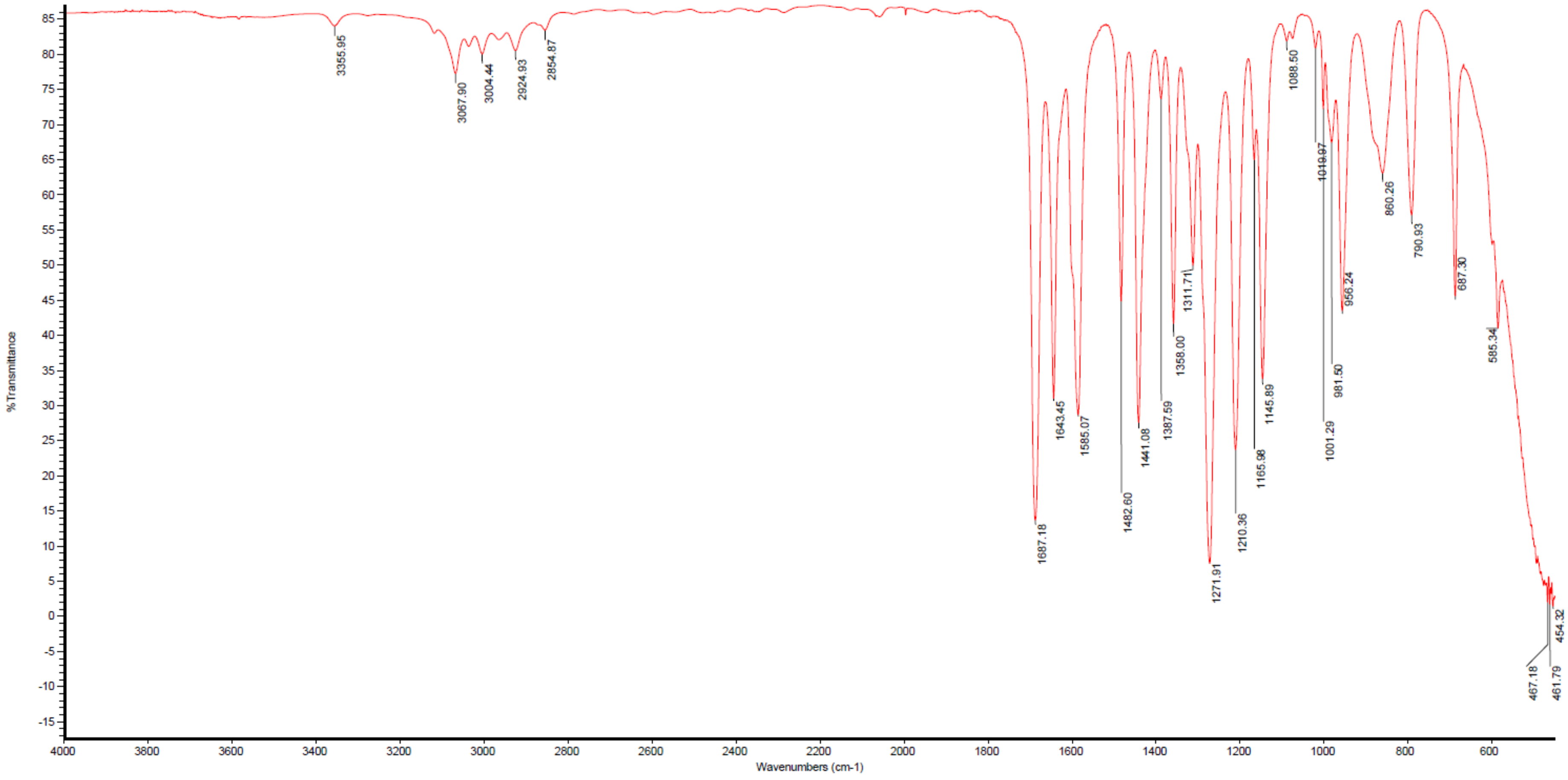

^{1}H NMR (500 MHz, Chloroform-*d*) of 1-(2,3-dihydrobenzofuran-4-yl)ethan-1-one (**4**)

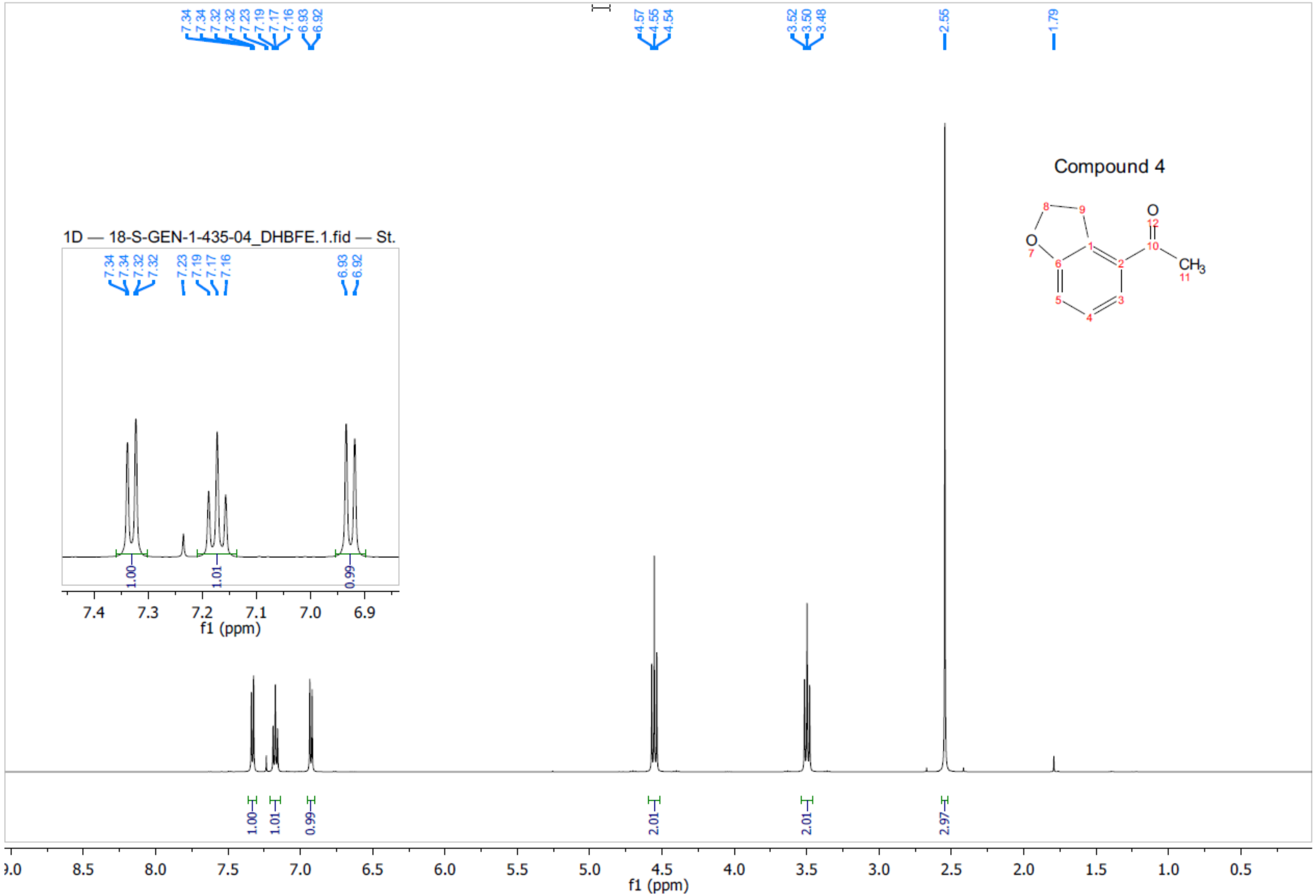

$^{13}C\{^1H\}$ NMR (125 MHz, Chloroform-*d*) of 1-(2,3-dihydrobenzofuran-4-yl)ethan-1-one (**4**)

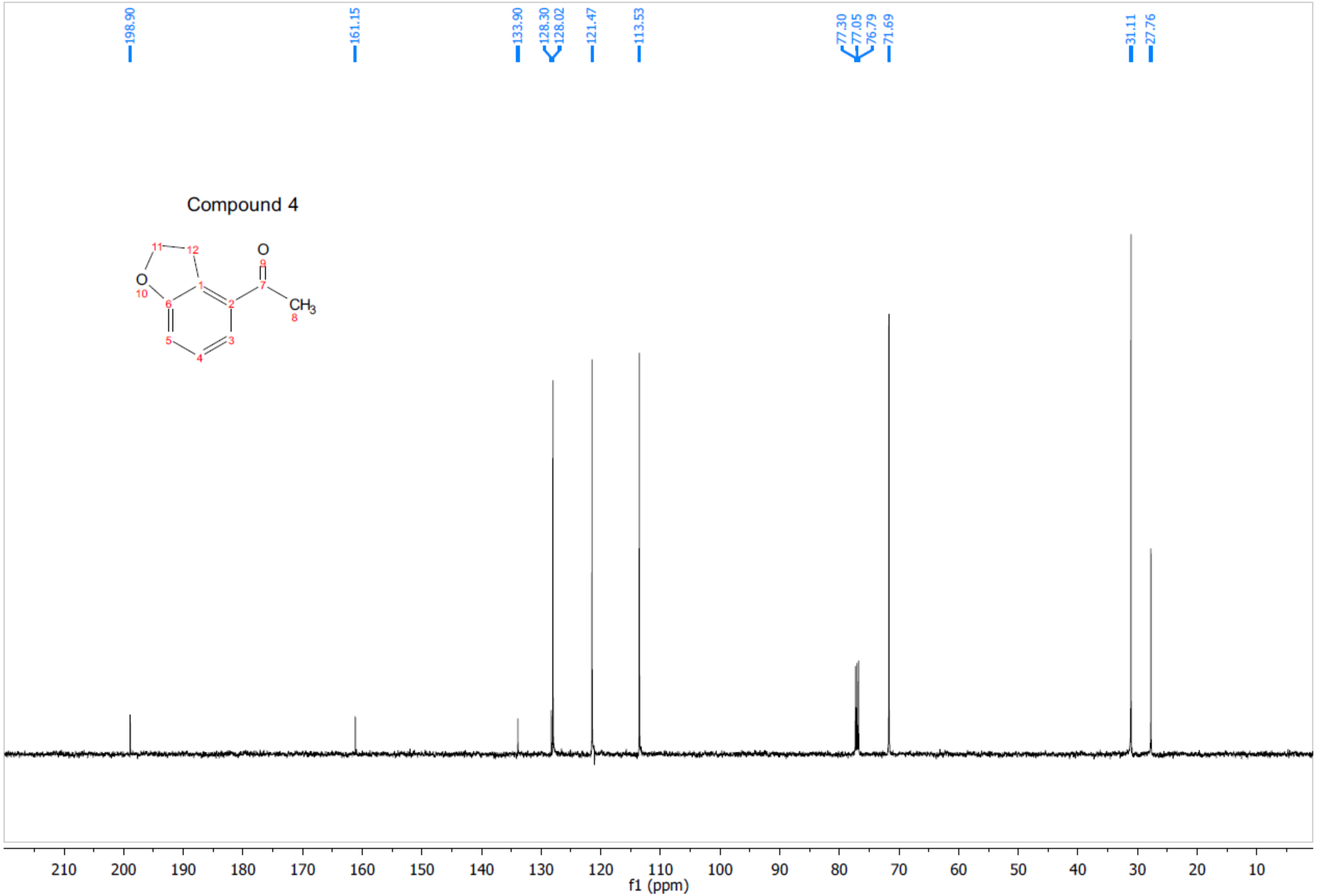

IR spectra of 1-(2,3-dihydrobenzofuran-4-yl)ethan-1-one (**4**)

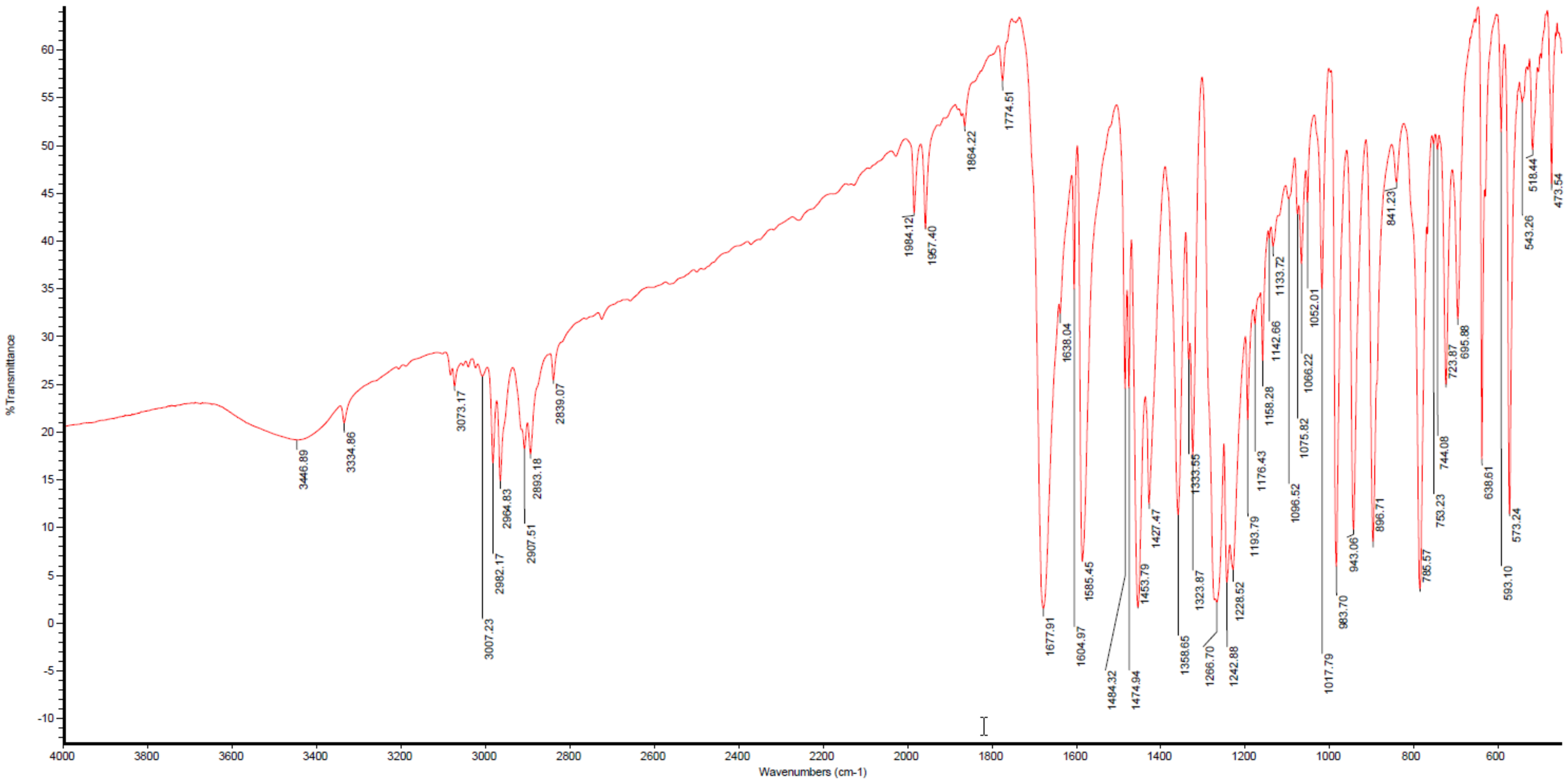

DSC thermogram of 1-(2,3-dihydrobenzofuran-4-yl)ethan-1-one (**4**)

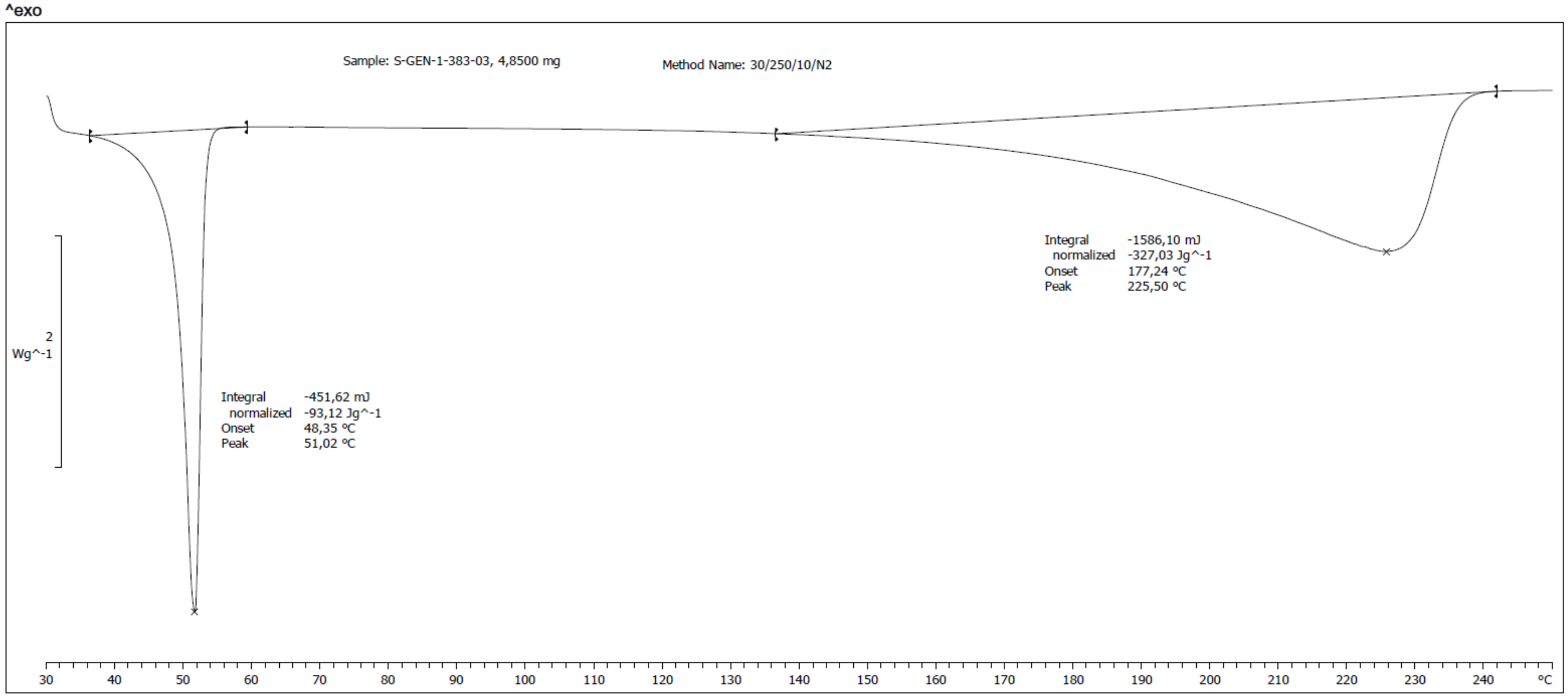

^{1}H NMR (500 MHz, Chloroform-*d*) of 1,2,6,7-tetrahydro-8*H*-indeno[5,4-*b*]furan-8-one (**5**)

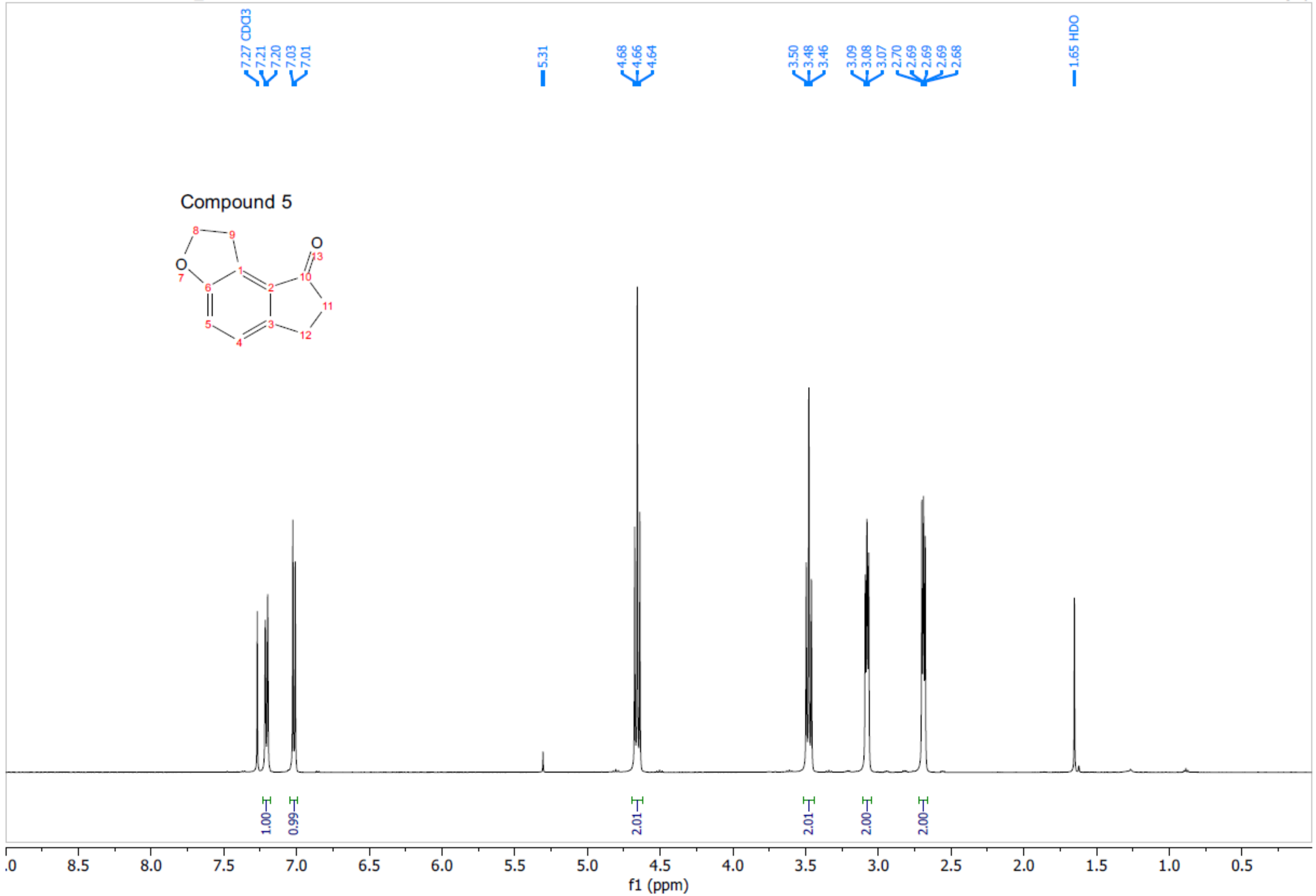

^{13}C{^{1}H} NMR (125 MHz, Chloroform-*d*) of 1,2,6,7-tetrahydro-8*H*-indeno[5,4-*b*]furan-8-one (**5**)

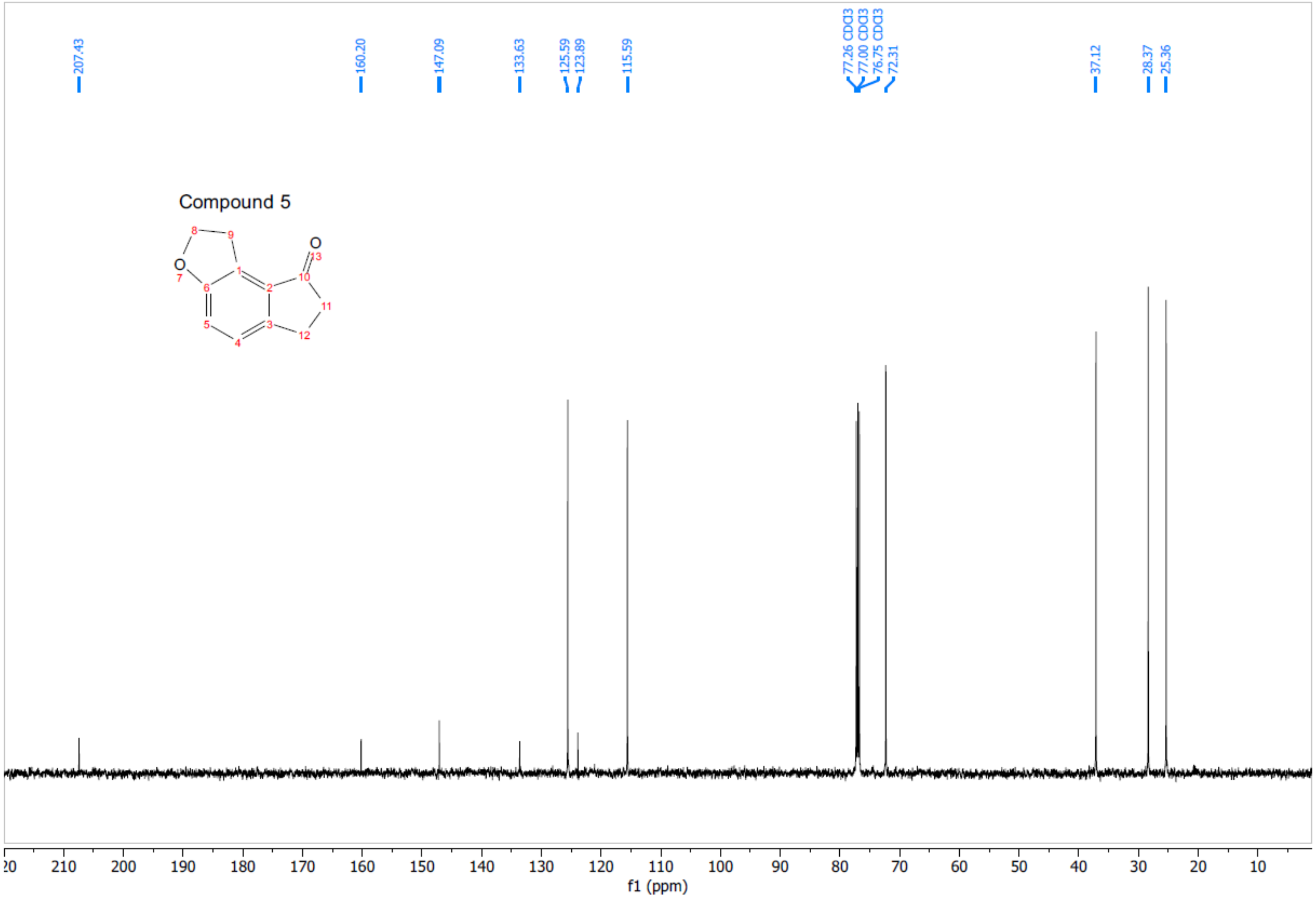

IR spectra of 1,2,6,7-tetrahydro-8*H*-indeno[5,4-*b*]furan-8-one (**5**)

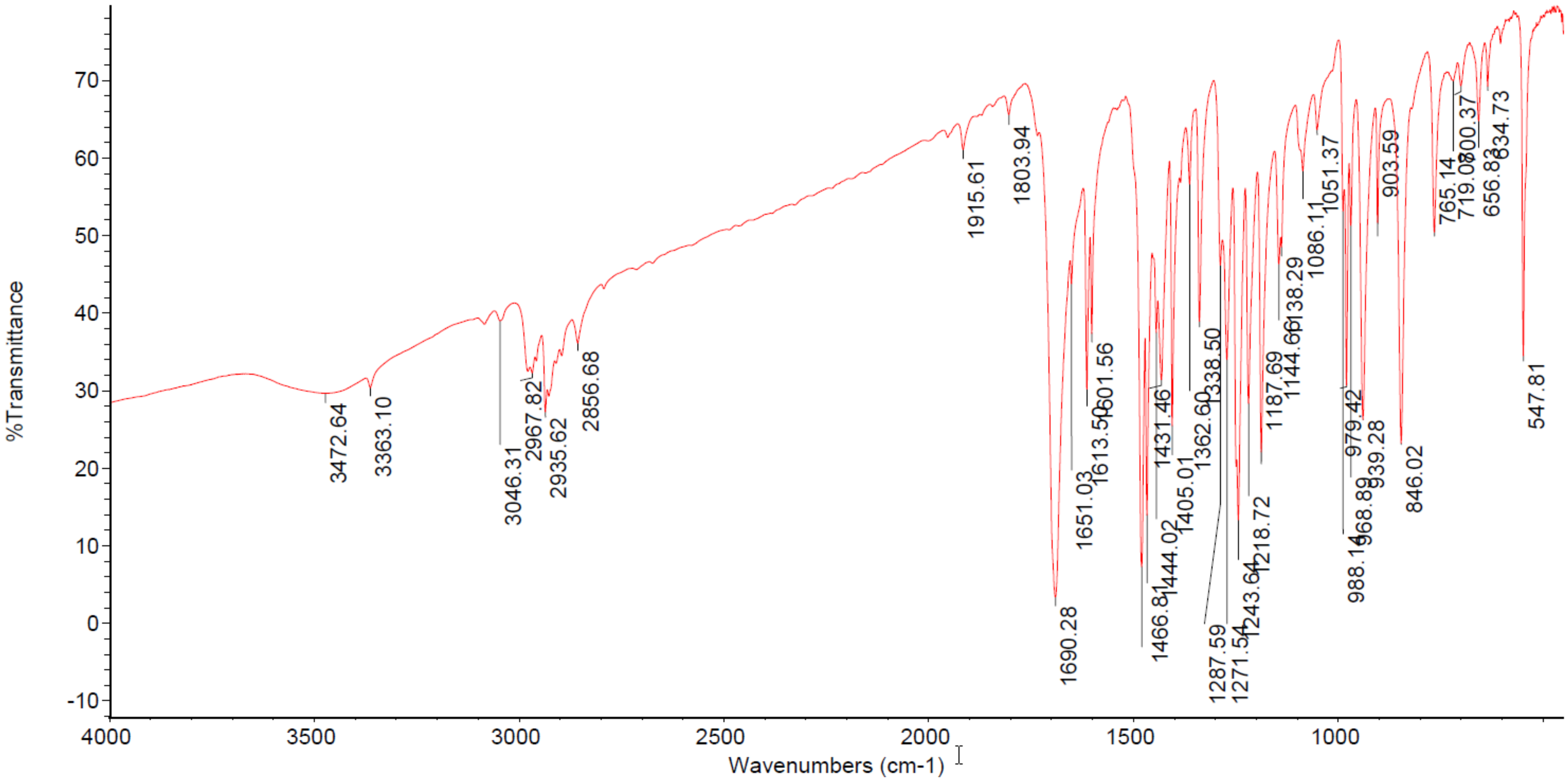

DSC thermogram of 1,2,6,7-tetrahydro-8*H*-indeno[5,4-*b*]furan-8-one (**5**)

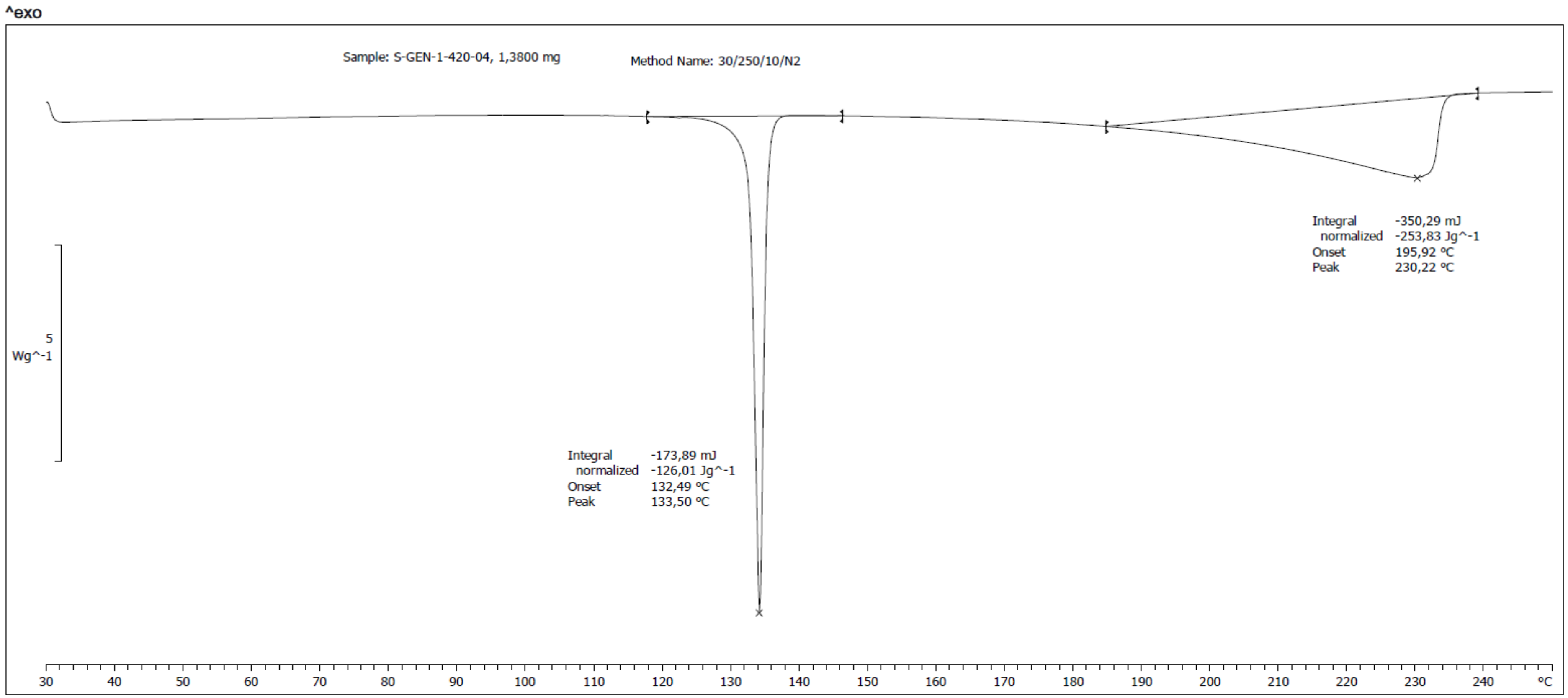

^{1}H NMR (500 MHz, Chloroform-*d*) of (*E*)-2-(1,2,6,7-tetrahydro-8*H*-indeno[5,4-*b*]furan-8-ylidene)acetonitrile (**7**)

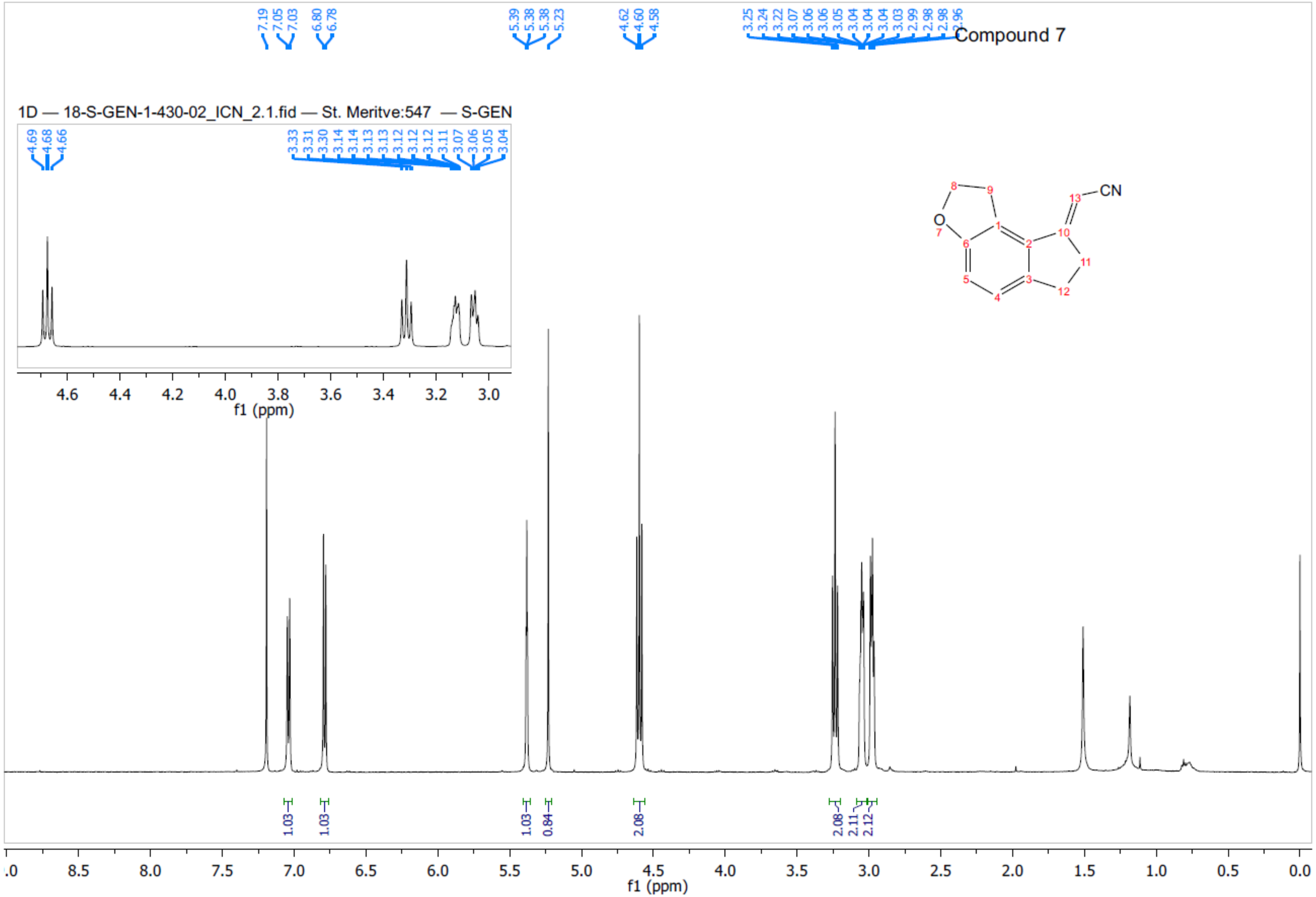

$^{13}C\{^1H\}$ NMR (125 MHz, Chloroform-*d*) of (*E*)-2-(1,2,6,7-tetrahydro-8*H*-indeno[5,4-*b*]furan-8-ylidene)acetonitrile (**7**)

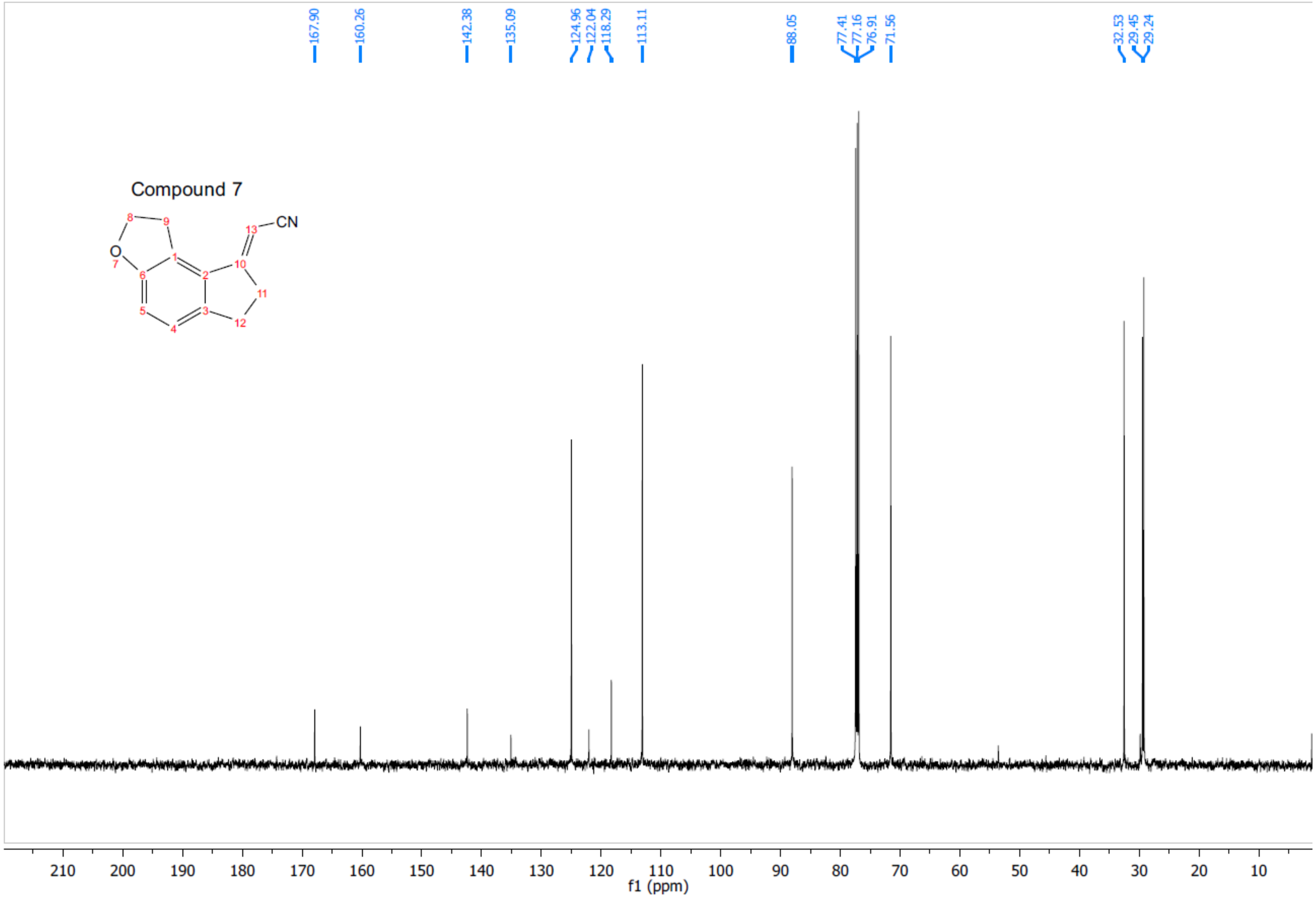

IR spectra of (*E*)-2-(1,2,6,7-tetrahydro-8*H*-indeno[5,4-*b*]furan-8-ylidene)acetonitrile (**7**)

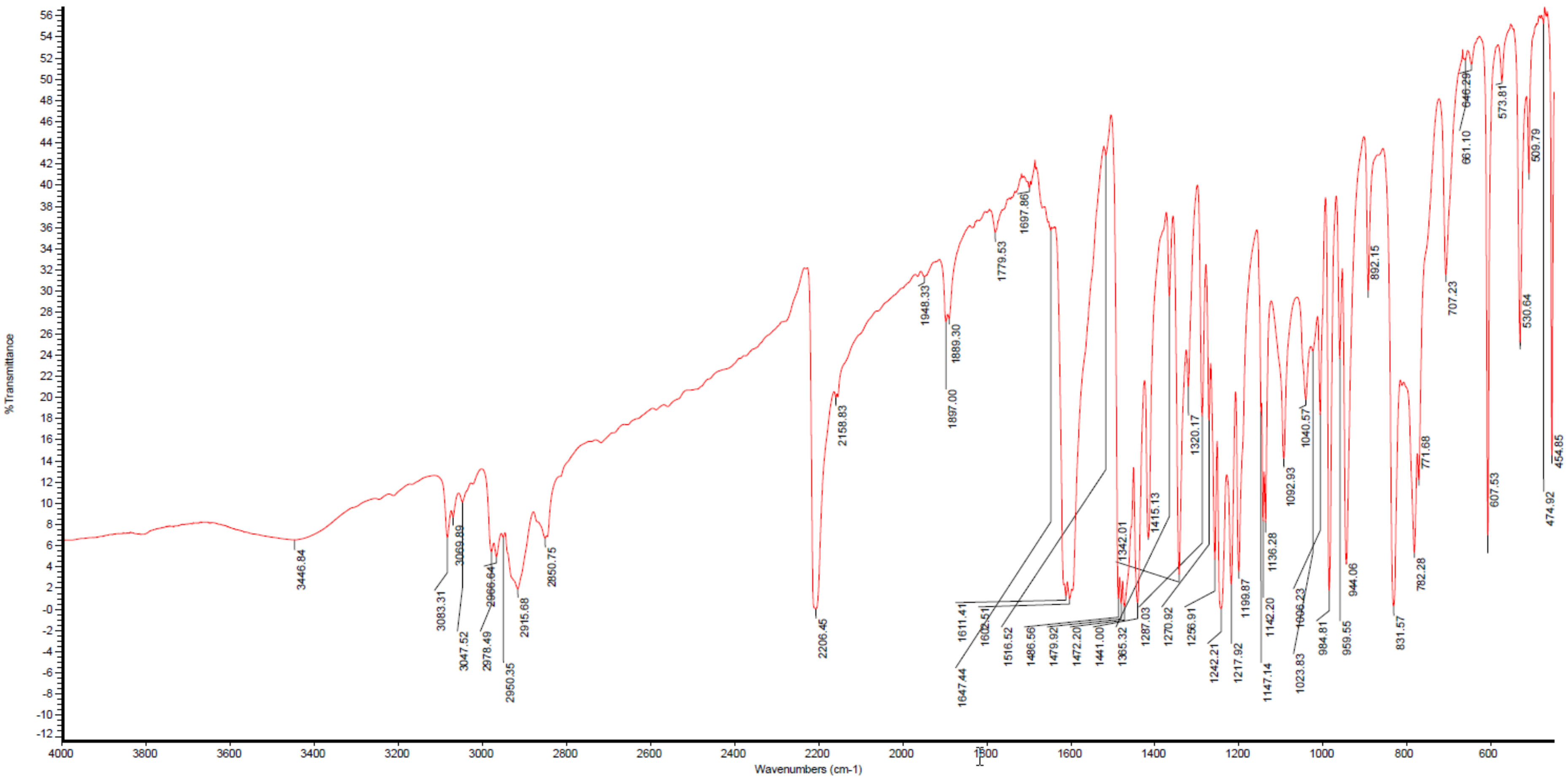

DSC thermogram of (*E*)-2-(1,2,6,7-tetrahydro-8*H*-indeno[5,4-*b*]furan-8-ylidene)acetonitrile (**7**)

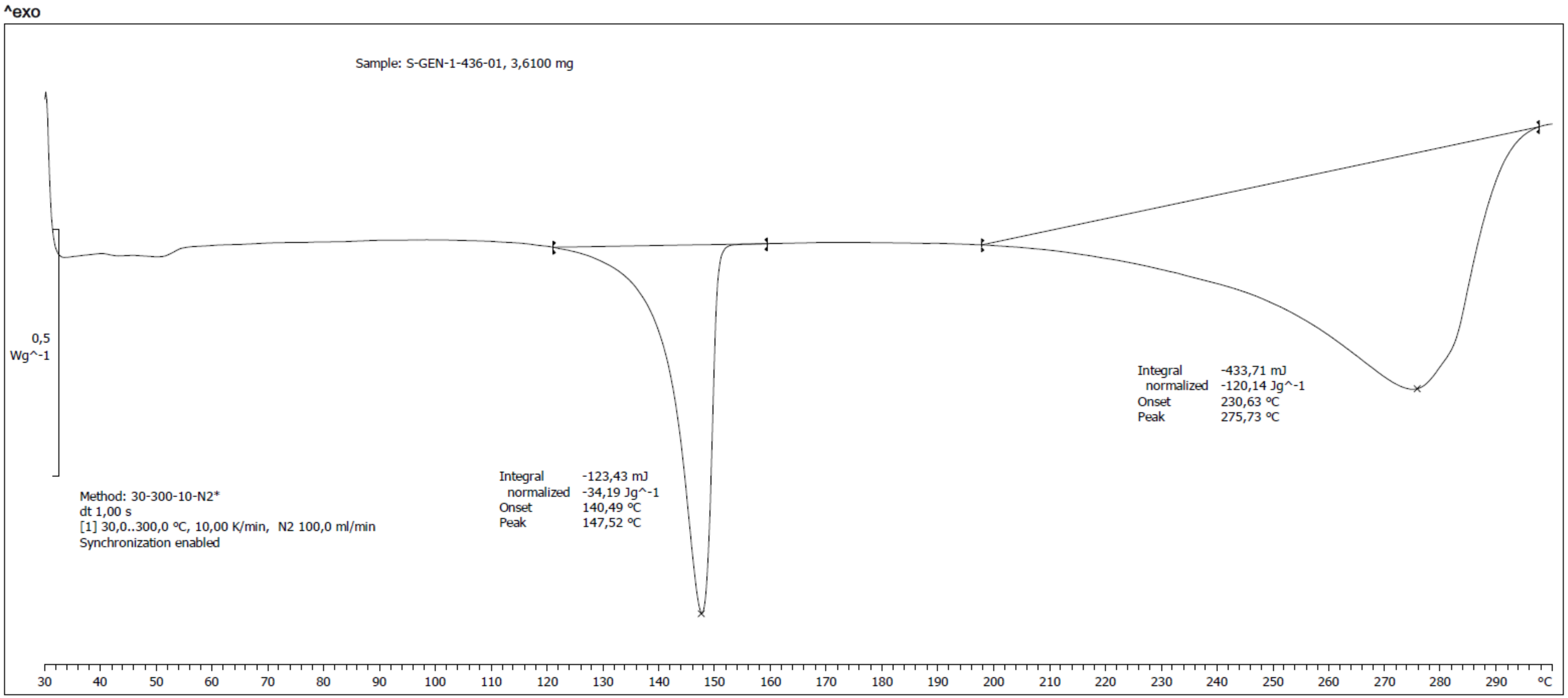

^{1}H NMR (500 MHz, Chloroform-*d*) of (*S*)-2-(1,6,7,8-tetrahydro-2*H*-indeno[5,4-*b*]furan-8-yl)acetonitrile (**8**)

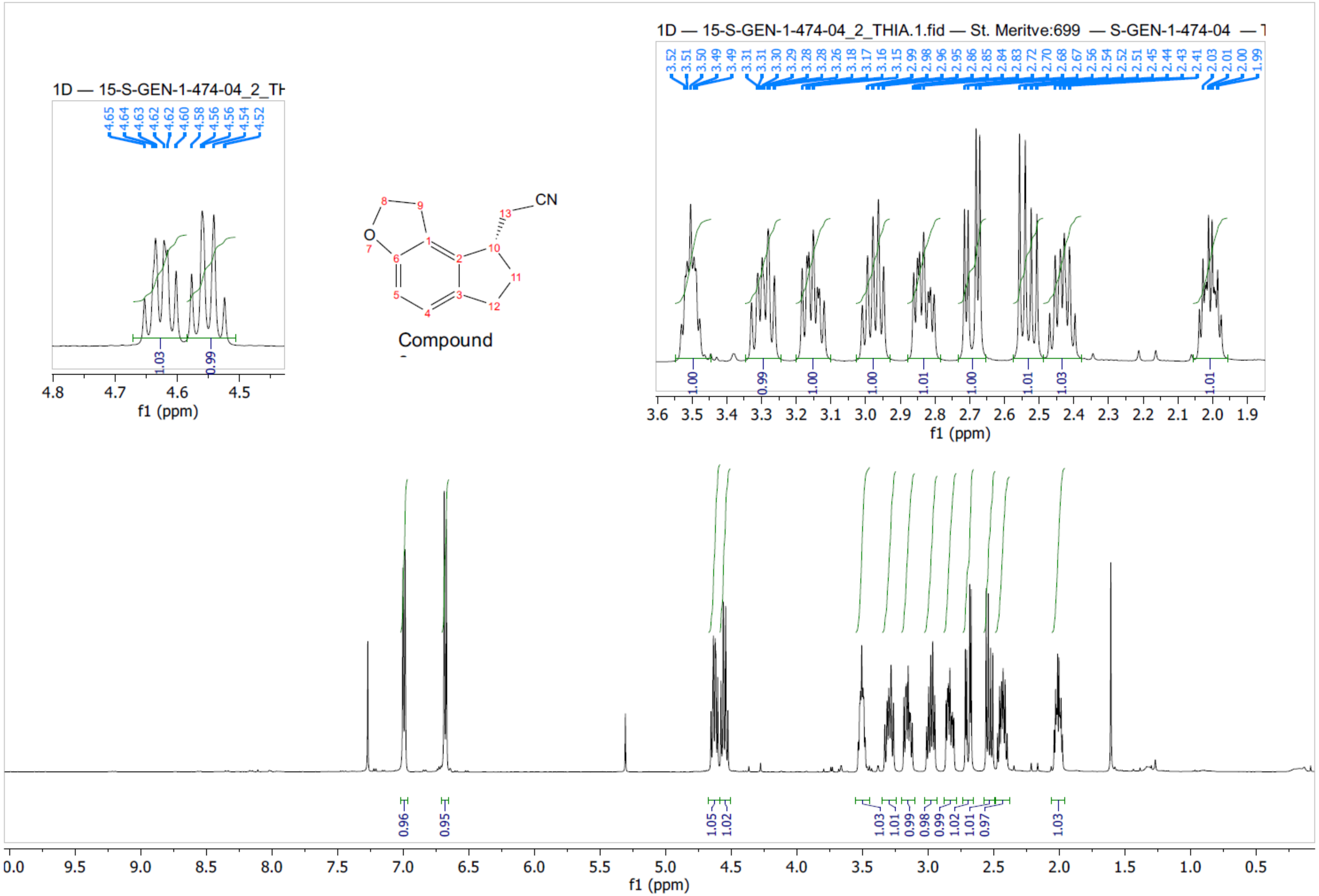

$^{13}C\{^{1}H\}$ NMR (125 MHz, Chloroform-*d*) of (*S*)-2-(1,6,7,8-tetrahydro-2*H*-indeno[5,4-*b*]furan-8-yl)acetonitrile (**8**)

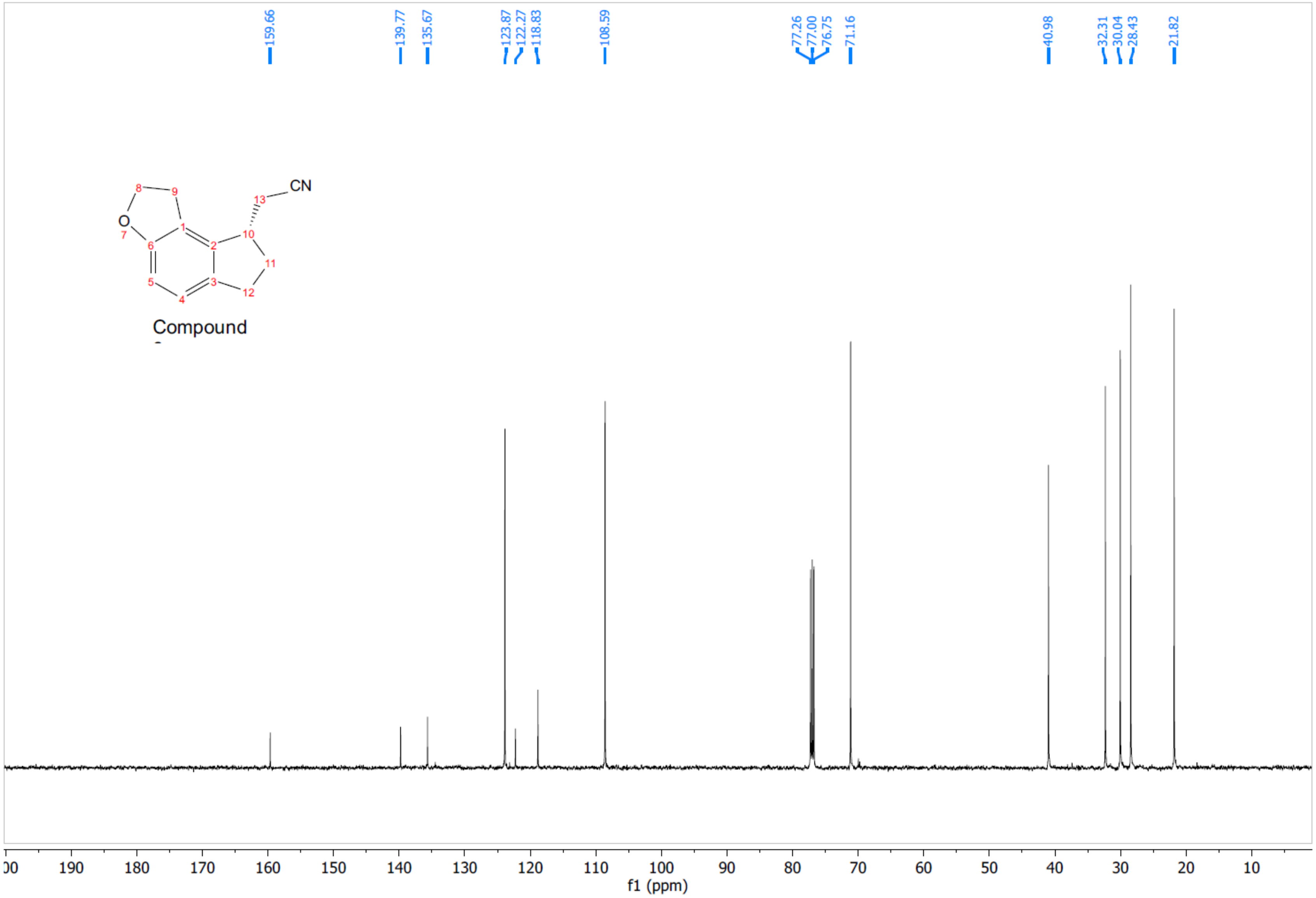

IR spectra of (*S*)-2-(1,6,7,8-tetrahydro-2*H*-indeno[5,4-*b*]furan-8-yl)acetonitrile (**8**)

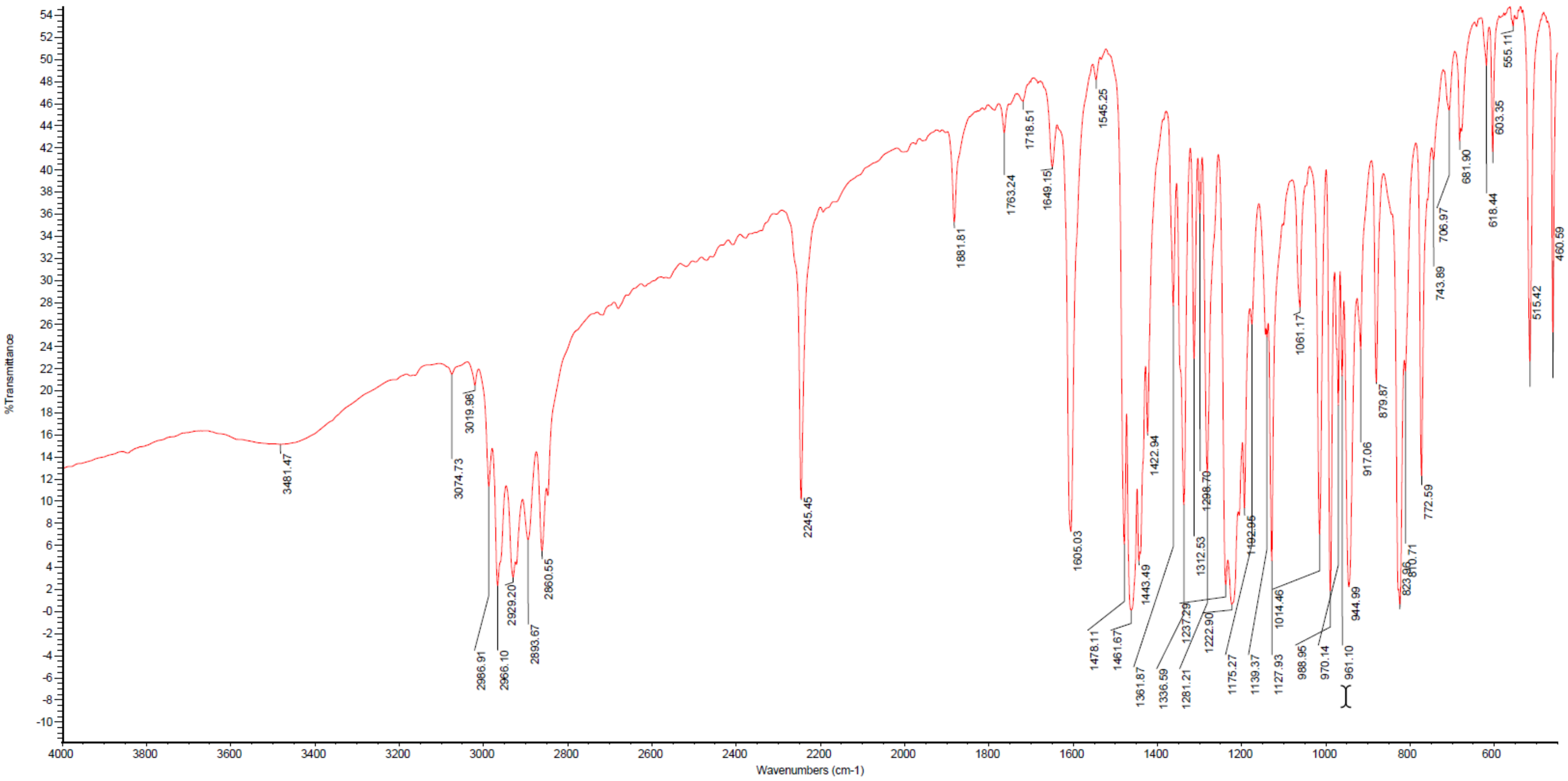

DSC thermogram of (*S*)-2-(1,6,7,8-tetrahydro-2*H*-indeno[5,4-*b*]furan-8-yl)acetonitrile (**8**)

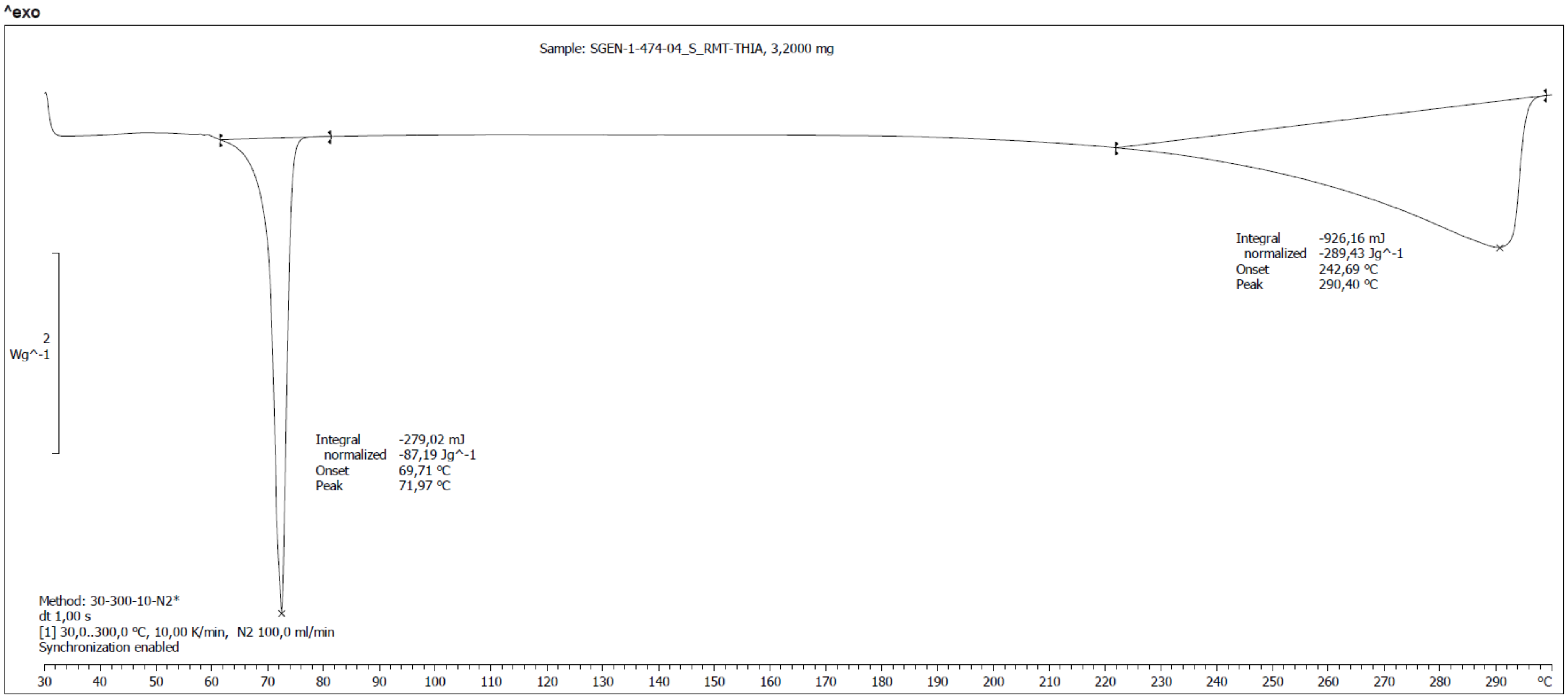

^{1}H NMR (500 MHz, Chloroform-*d*) of ramelteon (**9**)

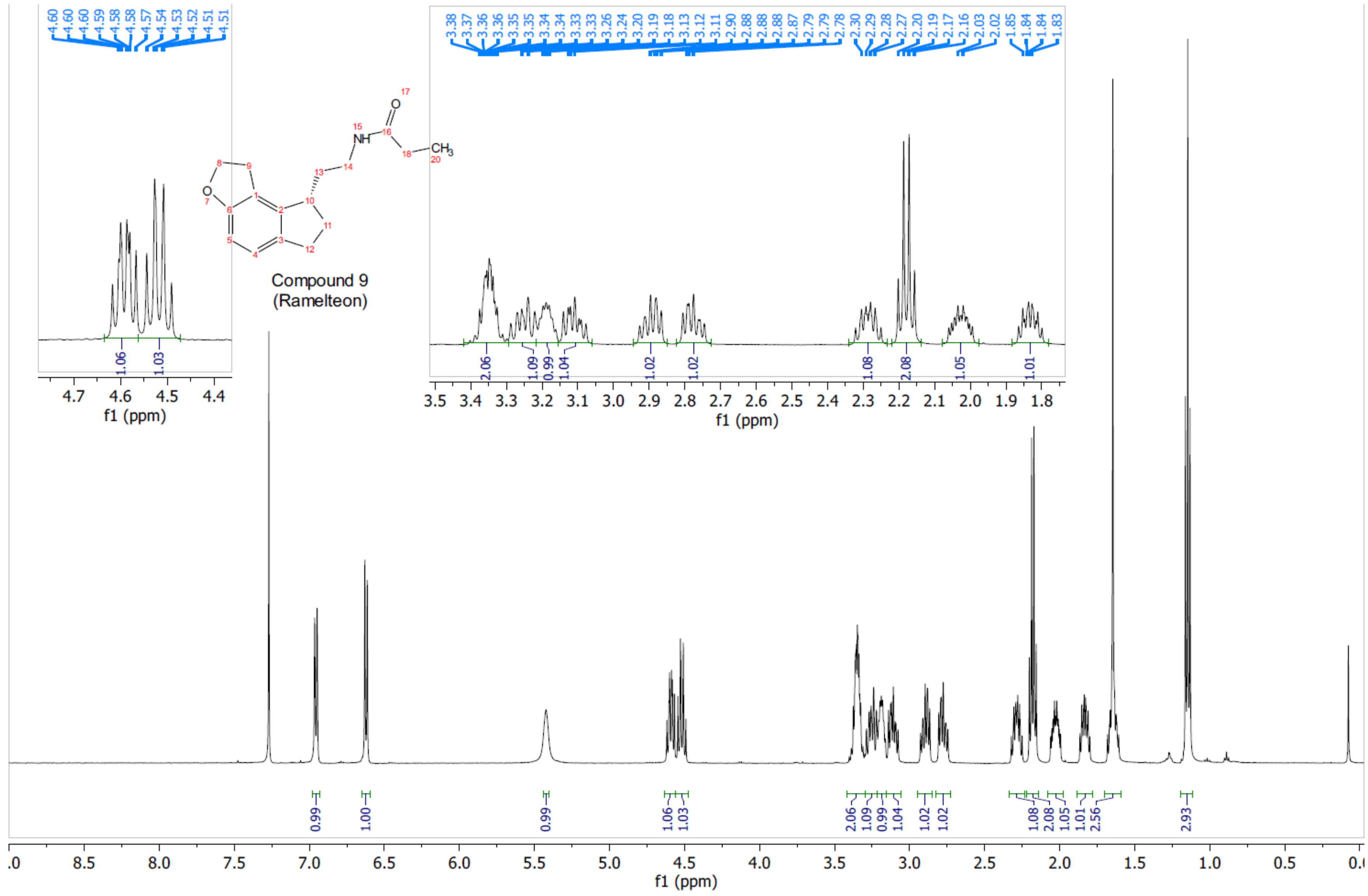

$^{13}C\{^1H\}$ NMR (125 MHz, Chloroform-*d*) of ramelteon (**9**)

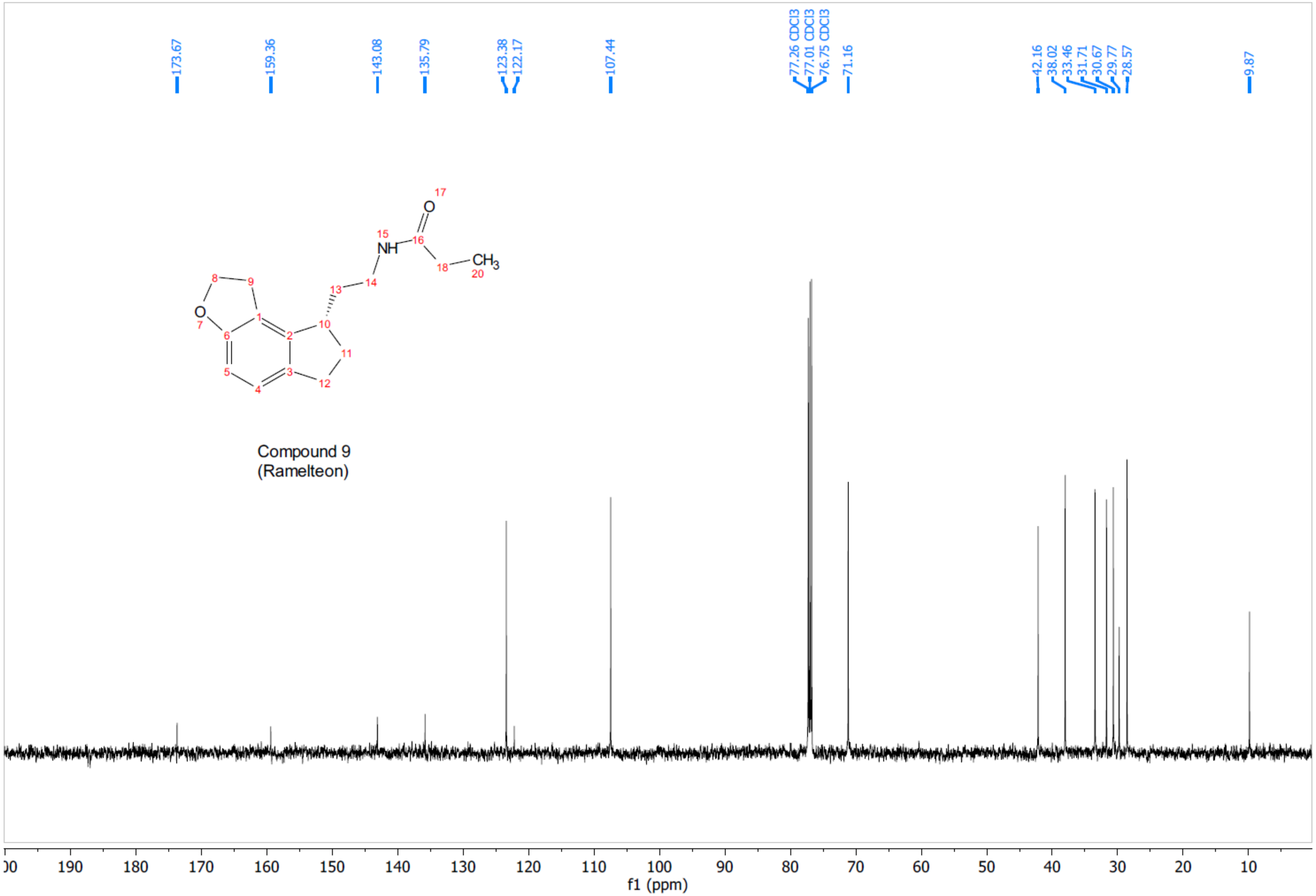

IR spectra of ramelteon (**9**)

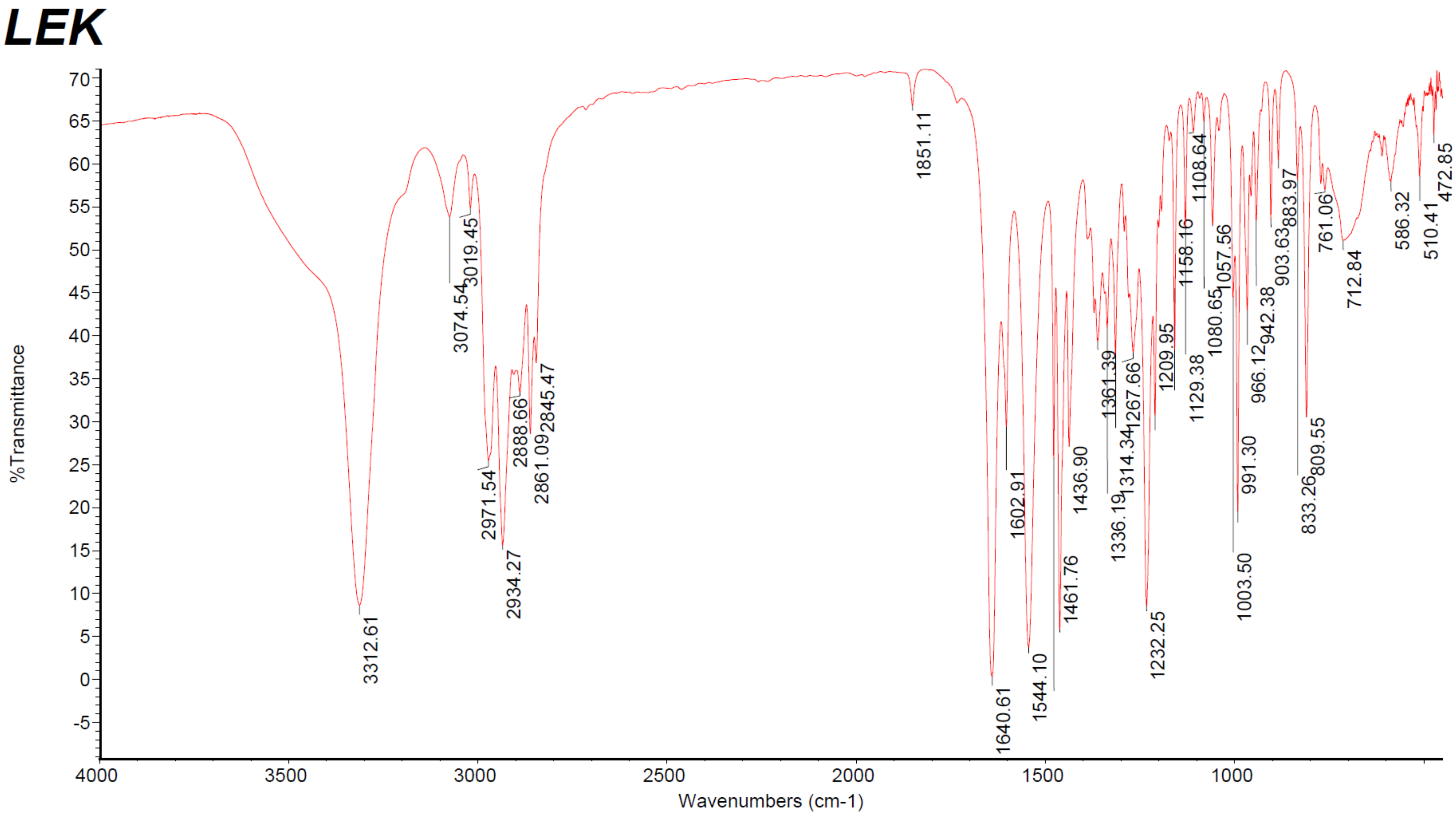

DSC of ramelteon (**9**)

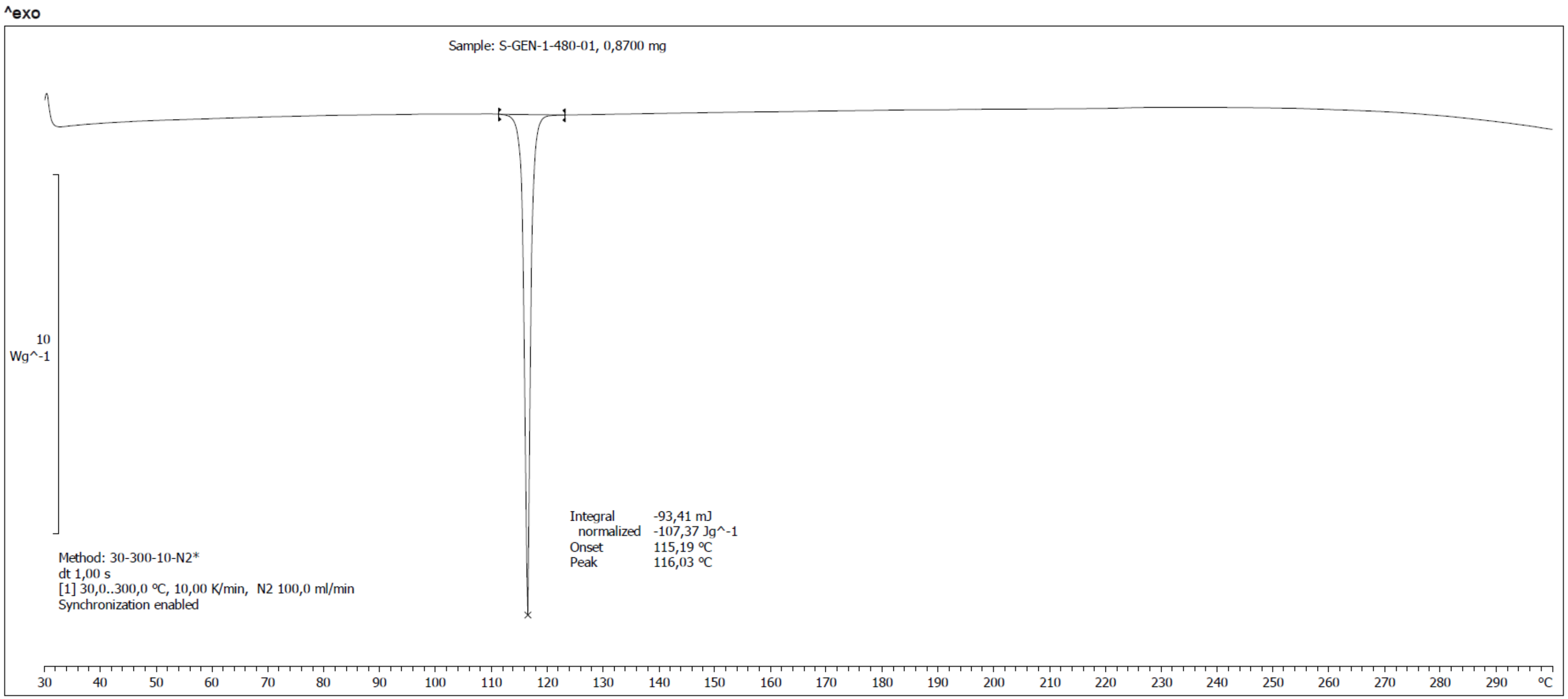

Chiral HPLC of (*S*)-2-(1,6,7,8-tetrahydro-2*H*-indeno[5,4-*b*]furan-8-yl)acetonitrile (**8**)

Principle: Normal phase HPLC determination on chiral column

Standards: 1. Characteristic sample of (*S*)-**8**

Reagents:
1. *n*-heptane, C_7H_{16}
2. 2-propanol, C_3H_7OH

Chromatographic conditions:

1.	Mobile phase:	*n*-heptane / 2-propanol = 95/5 (v/v)
2.	Column:	Chiralpak AD-H, 250 × 4.6 mm
3.	temperature of the column:	30 °C
4.	Flow rate:	1.0 mL/min
5.	Wavelength:	230 nm
6.	Injection volume:	10 µL

Solvent: *n*-heptane / 2-propanol = 90/10 (v/v)

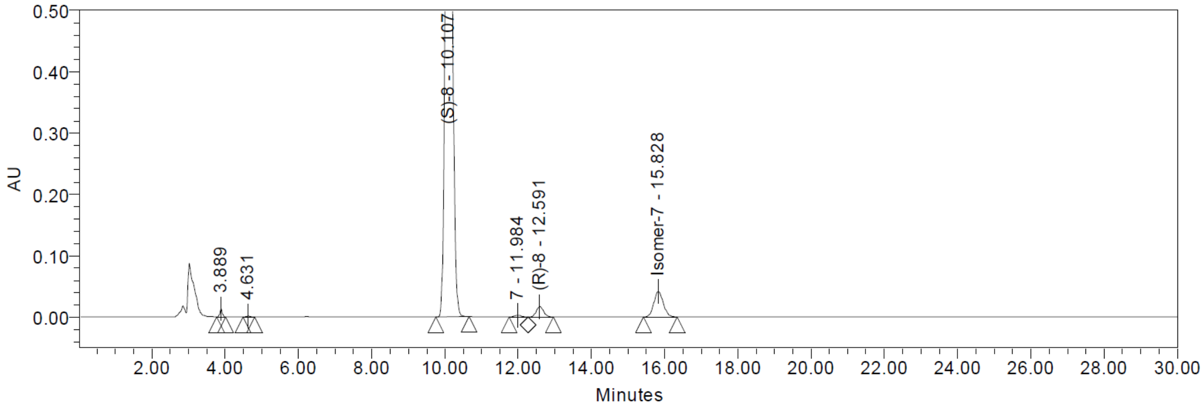

SampleName S-GEN-1-474-04; Vial 3; Injection 1; Date Acquired Monday, June 14, 2021 9:06:34 AM CEST; Date Processed Monday July 5, 2021 7:35:58 AM CEST

Sample Name: S-GEN-1-474-04

	Vial	Name	Retention Time (min)	Area (µV*sec)	% Area	EP s/n	Resolution	USP Tailing
1	3		3.889	59433	0.36			1.112829e+000
2	3		4.631	9969	0.06		4.7	1.053997e+000
3	3	(S)-8	10.107	15468918	93.48		21.8	1.056487e+000
4	3	7	11.984	33567	0.20		5.8	1.080847e+000
5	3	(R)-8	12.591	232781	1.41		1.7	1.074453e+000
6	3	Isomer-7	15.828	742284	4.49		7.7	1.089658e+000
Mean								
% RSD								
Sum					100.00			

$$ee_{\,(S)\text{-}8} = \frac{\text{Area\%}_{\,(S)\text{-}8} - \text{Area\%}_{\,(R)\text{-}8}}{\text{Area\%}_{\,(S)\text{-}8} + \text{Area\%}_{\,(R)\text{-}8}} \cdot 100\% = \frac{93.48\% - 1.41\%}{93.48\% + 1.41\%} \cdot 100\% = \frac{92.07\%}{94.98\%} \cdot 100\% = 96.9\%$$

Chiral HPLC of ramelteon (9)

Method used from reference 37: Patil, S. D.; Khandekar, N.; Kasawar, G. B.; Shaikh, K. A. Enantiomeric Separation of a Melatonin Agonist Ramelteon Using Amylose-Based Chiral Stationary Phase. *Arabian J. Chem*. **2013**, *6*, 103–109.

Chromatogram of Reference Material - Primary Standard obtained from LGC Standards GmbH.

Catalogue Number: LGCAMP3666.00-11; Lot Number: 107226, Release Date: Luckenwalde, March 2017

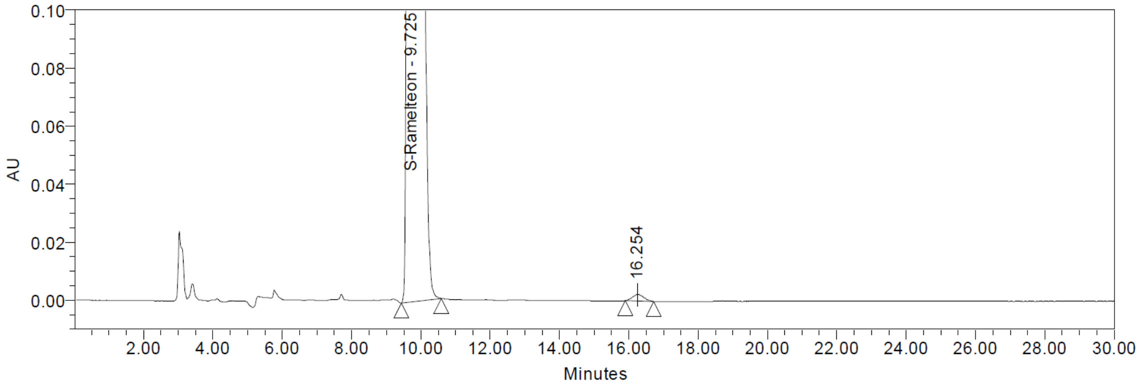

SampleName Ramelteon std.; Vial 12; Injection 1; Date Acquired Wednesday, June 30, 2021 2:46:07 PM CEST; Date Processed Thursday, July 1, 2021 6:27:49 AM CEST

Sample Name: Ramelteon std.

	Vial	Name	Retention Time (min)	Area (µV*sec)	% Area	EP s/n	Resolution	USP Tailing
1	12	S-Ramelteon	9.725	33431187	99.85			1.818212e+000
2	12	R-Ramelteon	13.900					
3	12		16.254	50918	0.15		11.5	1.146937e+000
Mean								
% RSD								
Sum					100.00			

Chromatogram of synthesised sample of ramelteon according to the procedure described in this article:

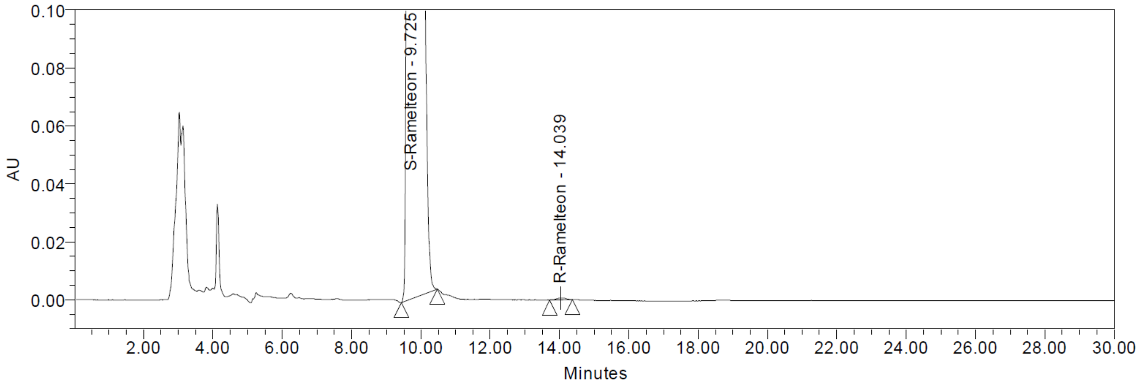

SampleName S-GEN-1-480-01; Vial 11; Injection 1; Date Acquired Wednesday, June 30, 2021 2:15:09 PM CEST; Date Processed Thursday, July 1, 2021 6:27:26 AM CEST

Sample Name: S-GEN-1-480-01

	Vial	Name	Retention Time (min)	Area (µV*sec)	% Area	EP s/n	Resolution	USP Tailing
1	11	S-Ramelteon	9.725	32441583	99.96			1.804053e+000
2	11	R-Ramelteon	14.039	12315	0.04		8.5	1.023566e+000
Mean								
% RSD								
Sum					100.00			

$$ee_{(S)\text{-}\mathbf{9}} = \frac{\text{Area\%}_{(S)\text{-}\mathbf{9}} - \text{Area\%}_{(R)\text{-}\mathbf{9}}}{\text{Area\%}_{(S)\text{-}\mathbf{9}} + \text{Area\%}_{(R)\text{-}\mathbf{9}}} \cdot 100\% = \frac{99.96\% - 0.04\%}{99.96\% + 0.04\%} \cdot 100\% = \frac{99.92\%}{100\%} \cdot 100\% = 99.9\%$$
